# A New Class of β–Pyrrolidino-1,2,3-Triazole Derivatives as β-Adrenergic Receptor Inhibitors: Synthesis, Pharmacological, and Docking Studies

**DOI:** 10.3390/molecules24193501

**Published:** 2019-09-26

**Authors:** Kaliyappan Easwaramoorthi, Jeya A. Rajendran, Kella Chennakesava Rao, Chandrasekar Balachandran, Yuvaraj Arun, Sakkarapalayam M. Mahalingam, Natarajan Arumugam, Abdulrahman I. Almansour, Raju Suresh Kumar, Dhaifallah M. Al-thamili, Shin Aoki

**Affiliations:** 1 Department of Chemistry, Loyola College, Chennai 600034, TN, India; kemysv.2018@gmail.com; 2 R&D Centre, Malladi Drugs & Pharmaceuticals Ltd., Chennai 600124, TN, India; kcrao2009@gmail.com; 3 Organic & Bioorganic Chemistry Laboratory, CSIR-Central Leather Research Institute, Adyar, Chennai 600020, TN, India; arun.y.chem@gmail.com; 4 Faculty of Pharmaceutical Sciences, Tokyo University of Science, 2641 Yamazaki, Noda 278-8510, Japan; balaeri09@gmail.com (C.B.); shinaoki@rs.noda.tus.ac.jp (S.A.); 5 Department of Chemistry, SRM institute of Science and Technology, Kattankulathur, Kancheepuram 603203, India; mahalins@srmist.edu.in; 6 Department of Chemistry, College of Science, King Saud University, P.O. Box 2455, Riyadh 11451, Saudi Arabia; anatarajan@ksu.edu.sa (N.A.); almansor@ksu.edu.sa (A.I.A.); sraju@ksu.edu.sa (R.S.K.);; 7 Research Institute of Science and Technology, Tokyo University of Science, 2641 Yamazaki, Noda 278-8510, Japan

**Keywords:** β–pyrrolidino-1,2,3-triazole, β-adrenoceptors, antimicrobial activity, anticancer activity, docking studies

## Abstract

New 1,4-disubstituted β-pyrrolidino-1,2,3-triazoles were synthesized using a reusable copper-iodide-doped neutral alumina catalyst. Synthesis of diversely substituted triazoles and recyclability of CuI catalyst explains the broad scope of this protocol. The synthesized compounds were screened for their antimicrobial and anticancer properties. Most of the compounds showed significant antimicrobial activities against all the tested microorganisms compared to standard drugs. Furthermore, compounds **5a**, **5e**, **5g**, **5h**, **5i**, and **5j** showed moderate to potent activities against A549 and HepG-2 cells. In addition, compounds **5g** and **5h** displayed potential cytotoxicity activity against A549 cells with IC_50_ values of 72 ± 3.21 and 58 ± 2.31 µM, respectively. The molecular docking study revealed that some of the synthesized compounds exhibited comparable binding as co-crystalized ligands with the DNA topoisomerase IV and anaplastic lymphoma kinase receptors.

## 1. Introduction

Cancer is a multi-factorial genetic disease whose development involves a multitude of genes [1]. Microarray analyses have identified thousands of genes that are over- or under-expressed in specific tumor samples. However, the use of more specific techniques, such as real-time quantitative polymerase chain reactions, is necessary to further investigate the involvement of each of these genes [2]. β-Adrenergic receptors transduce signals from catecholamines to the G protein, which through a cascade of chemical reactions, generates highly specific parallel signals with neuroendocrine functions in cells. Previous studies have established a relationship between the β_2_-adrenergic receptor and various aspects related to cancer. This receptor appears to be mainly associated with cell proliferation, apoptotic phenomena [3,4], chemotaxis, the development of metastasis [5,6], tumor growth, and angiogenesis [7].

**Figure 1:**
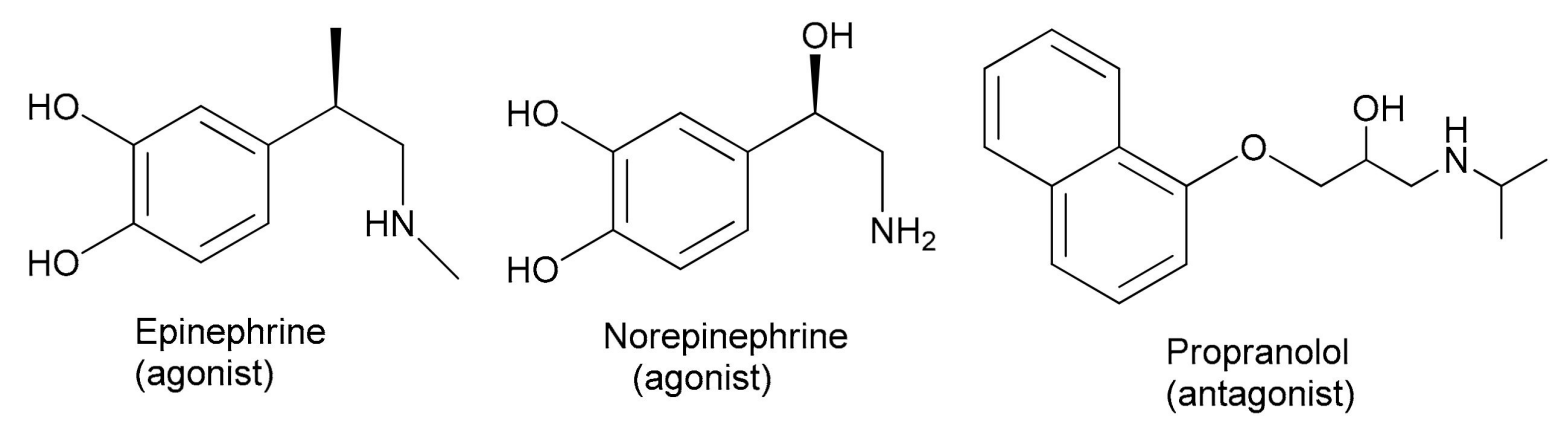
Structures of β-adrenergic receptor agonists and antagonist.

In their role as β-adrenergic receptor agonists, catecholamines and, in particular, epinephrine and norepinephrine are directly involved in increasing the metastatic capacity and apoptosis resistance of carcinogenic cells. β-Adrenergic receptors, in particular β_1_- and β_2_-receptors, have also been found to be expressed in colon cancer cell lines [8] and oral squamous cell carcinoma (OSCC) [9]. In OSCC, it appears that the over-expression of β_2_-adrenergic receptors in TCa8113 and ACC cells and the use of the β-adrenergic receptor agonist norepinephrine cause a potent mitogenic effect that increases tumor size, while the antagonist propranolol, in contrast, completely inhibits these receptors (Figure 1).

**Scheme 1:**
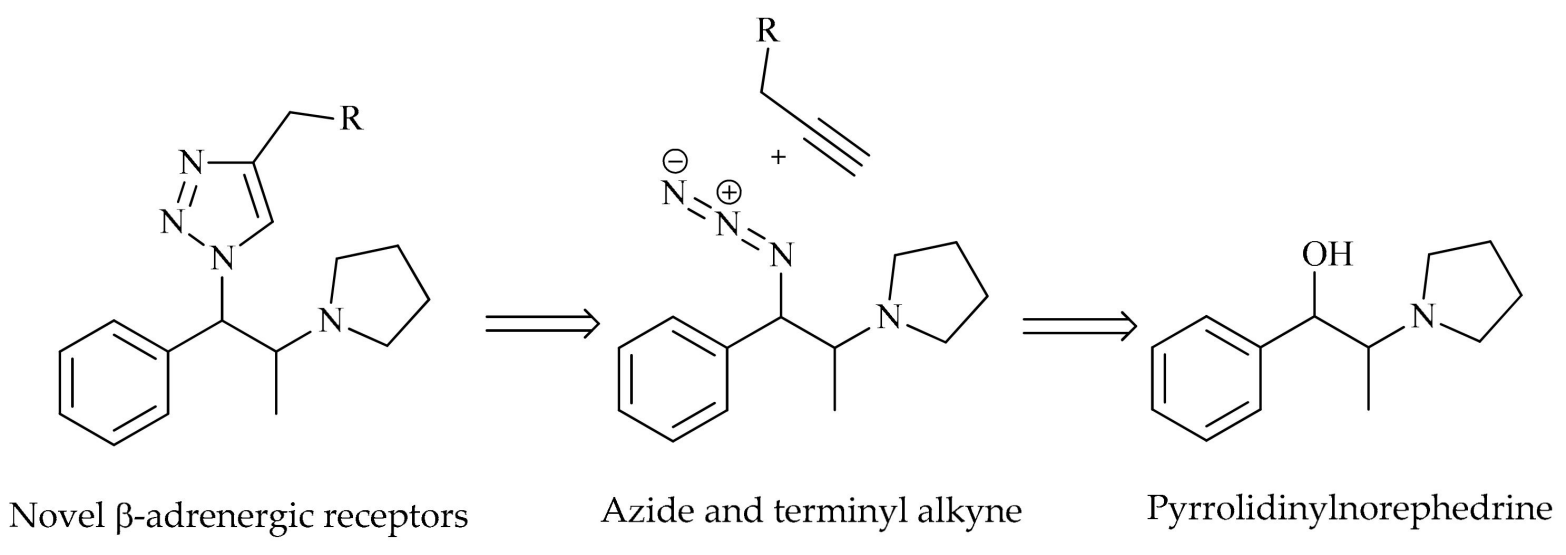
Design strategy for new β-adrenergic receptor inhibitors.

A recent in vivo study using colon cancer xenografts showed an association between nicotine and increased tumor size and vascularization that was attributable to an increase in the synthesis of COX-2 (Cyclooxygenase-2) prostaglandin E2, and VEGF (Vascular Endothelial Growth Factor) mediated by β-adrenergic receptors, particularly β2 receptors [9]. Therefore, the development of novel β-adrenergic receptor inhibitors possessing improved pharmaceutical profiles and reduced adverse effects is still urgently needed. Various potent pharmacological activities [10,11,12] of pyrrolidinyl triazoles including anticancer potential and acting as MPTP (Mitochondrial Permeability Transition Pore) blockers prompted us to explore 1,4-disubstituted β-pyrrolidino-1,2,3-triazoles as β-adrenergic receptor inhibitors. The synthesis was accomplished as described in Scheme 1 from pyrrolidinylnorephedrine using copper-catalyzed 1,3-dipolar cycloaddition with azide and terminal alkyne as an application of click chemistry [13,14].

## 2. Results and Discussions

### 2.1. Chemistry

**Scheme 2:**
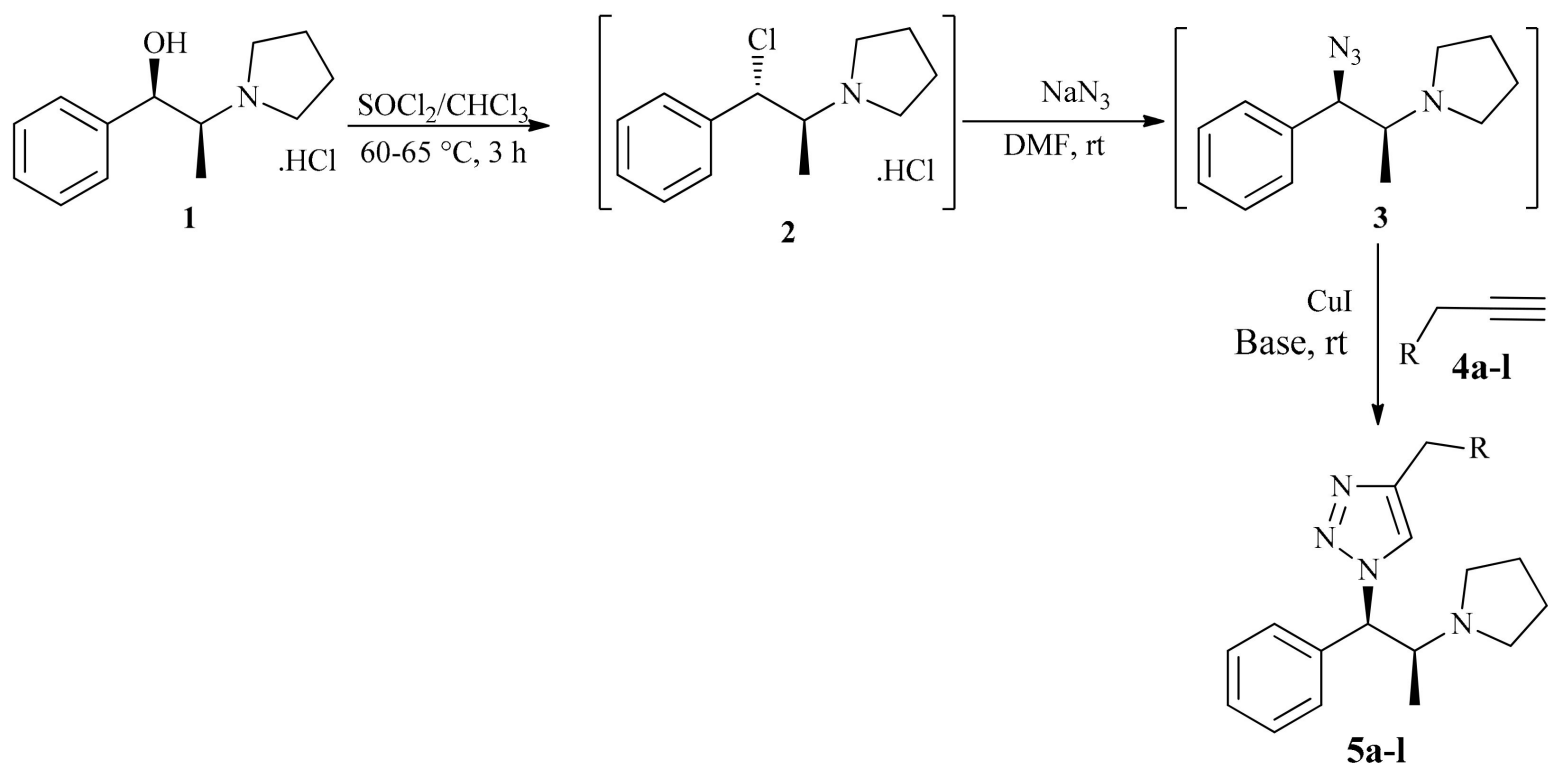
Synthesis of novel β-pyrrolidino-1,2,3-triazoles by CuAAC.

Our synthetic study began with a readily available starting precursor (1*R*,2*S*)-pyrrolidinylnorephedrine **1** (PNE) being subjected to sequence of transformations as outlined in Scheme 1. Thus, PNE **1** received treatment with thionyl chloride under refluxing condition in chloroform and was converted into its chloro derivative **2**, which upon treatment with sodium azide in N,N-dimethylformamide (DMF) at ambient temperature for 12 h, produced the azide **3** in quantitative yield (Scheme 1). Finally, the terminal alkynes **4a**–**l** (except **4a**–**b** and **4f** [15]) were allowed to react with azide **3** employing copper-catalyzed 1,3-dipolar cycloaddition (also known as CuAAC—copper catalyzed azide-alkyne-cycloaddition), which produced the target novel 1,4-disubstituted β-pyrrolidino-1,2,3-triazoles **5a-l** as an application of click chemistry (Scheme 2).

An optimization study was performed using propargyl alcohol **4a** as a terminal alkyne. The yield of **5a** was considered as a target in all experiments during this optimization study. Various reaction conditions, such as the concentration of copper iodide, supported on alumina, various solvents, and temperature, have been studied to establish the best condition for the formation of novel 1,4-disubstituted β-amino-1,2,3-triazole **5a** in good yield (Table 1).

**Figure 2:**
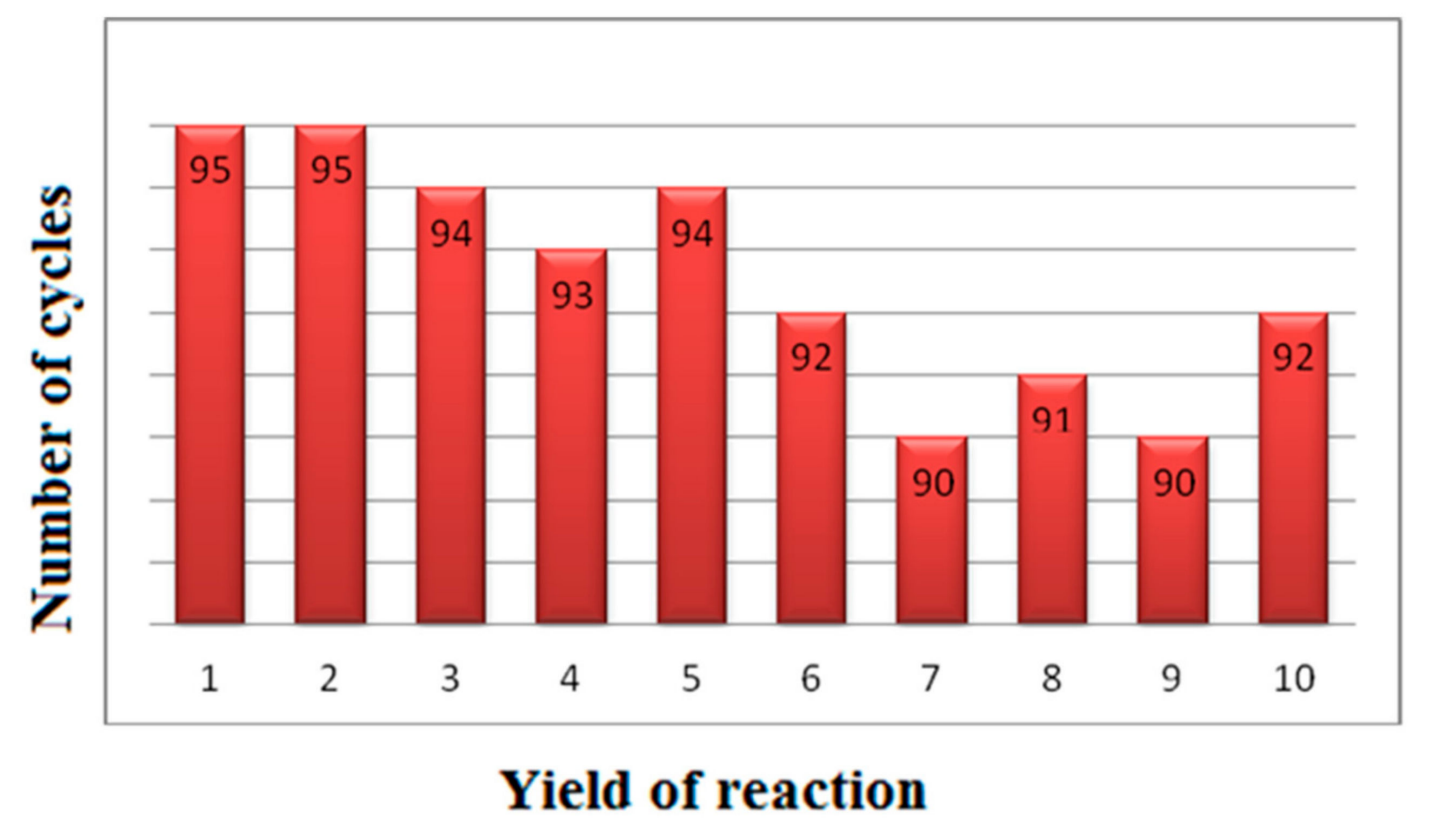
Re-usage of catalyst: Number of cycles vs. yield of reaction, **5a.**

Bases such as diisopropylethylamine (DIPEA), triethylamine (TEA), and potassium hydroxide were used and better yields were noticed with DIPEA. Solvents such as various alcohols and tetrahydrofuran (THF) were used and good results were found with the usage of a mixture of solvent that consisted of alcohol (methanol or ethanol), THF, and water in an equimolar ratio. Among copper bromide and copper iodide as catalysts, the reaction proceeded well with copper iodide. From Table 1 and entry 11, it was understood that usage of 10% copper iodide/alumina (CuI/Al_2_O_3_) in 5 mol% produced compound **5a** with a 95% yield. Another advantage of the used catalyst is that it can be reusable without a loss of its efficiency. After each reaction, the catalyst was washed with methanol, dried at 100 °C, and reused. In our study, the catalyst was recycled 10 times and produced satisfactory yields in-between 90% and 95% (Figure 2). However, additional time was required for the completion of the reaction. The time required for the completion of the reaction for the first and tenth cycles was 8 h and 20 h, respectively.

Usage of CuI/Al_2_O_3_ in click chemistry has already been reported [16] under microwave treatment, which is not feasible at the industrial scale [17] and it is an expensive technique. From Table 1, it was concluded that the usage of 5 mol% of CuI/Al_2_O_3_ catalyst, DIPEA as a base, and a mixture of methanol/THF/water as a solvent system at 25 °C to 30 °C produced the required compound **5a** with a very good yield (95%). The same reaction condition was used for subsequent reactions (**5b**–**l, Table 2**).

For this activity, we used hydrochloride salt of (1*R*,2*S*)-pyrrolidinylnorephedrine **1 [18]**, which was synthesized from (1*R*,2*S*)-phenylpropnaolamine. Since the key starting material **1** is chiral, the final triazoles **5a**–**l** should also be chiral. During chlorination of **1** (Scheme 2), the inversion took place at the benzylic carbon followed by another inversion through an azidation reaction, which resulted in an overall retention of the configuration at the benzylic carbon. Analysis of the isolated compounds using chiral HPLC (High Performance Liquid Chromatography) shows that all compounds were chirally pure. The specific optical rotation was not measured since the solution of the compounds (1% concentration in methanol or chloroform) was quite dark due to the dark colors of the products (brown solids) and a zero reading was observed with the polarimeter.

Chiral HPLC analysis: Compound **5a** was prepared from two separate experiments as per the established method (Scheme 2). In the first experiment (1*R*,2*S*)-pyrrolidinylnorephedrine **1** was used and in the second experiment racemic-pyrrolidinylnorephedrine was used. The obtained compounds from both the experiments were analyzed using normal HPLC and chiral HPLC. In normal HPLC, only one peak was found for the compound, which was obtained from both the experiments at the same retention time (RT). However, in chiral HPLC, the compound obtained from the first experiment showed one major peak with an area% above 99%. The compound obtained from the second experiment showed two peaks using chiral HPLC with an equal intensity and the retention time of one peak was matched with the compound of the first experiment. This study indicated that the compound obtained from the first experiment was a chiral one with enantiomeric excess (ee) above 99% and the compound obtained from the second experiment was a racemic mixture (*vide infra*, Supplementary Materials).

The structures of the synthesized novel 1,4-disubstituted-β-pyrrolidino-1,2,3-triazoles **5a**–**l** (Table 2) were well established using FT-IR, ^1^H-NMR, ^13^C-NMR, and mass spectroscopy. As a representative case, the structural elucidation of compound **5h** is described here. In the IR spectrum (KBr) of **5h**, the medium bands at 3142, 3086, 3063, 2965, and 2905 cm^−1^ indicated stretching frequencies of aliphatic and aromatic C–H. The sharp band at 1701 cm^−1^ indicated the presence of ester carbonyl and the bands at 1599 and 1514 cm^−1^ confirmed benzenoid stretching frequencies. The bands at 1342 cm^−1^ and 1291 cm^−1^ confirmed the presence of C–N (aromatic) and C–O (aromatic ester) groups, respectively. Two characteristic bands at 1020 and 949 cm^−1^ represented C–H bending frequencies of the 1,2,3-triazole ring [19]. The presence of bands at 843 and 785 cm^−1^ indicated C–H bending frequencies of the trisubstituted phenyl ring and a band at 768 cm^−1^ represented a mono-substituted phenyl ring.

In the ^1^H-NMR spectrum of **5h**, the doublet signal at δ_H_ 1.02 ppm (*J* = 1.2 Hz) indicated the presence of three protons of the aliphatic CH_3_ group. A broad multiplet signal at δ_H_ 1.66 ppm showed four protons of two CH_2_′s of a pyrrolidinyl ring at the C_3_ and C_4_ carbons. Another broad multiplet signal at δ_H_ 2.59–2.61 ppm indicated four protons of two CH_2_ groups of a pyrrolidinyl ring at the C_2_ and C_5_ carbons. Two sharp singlet signals at δ_H_ 3.92 ppm and 3.94 ppm confirmed six protons of two OCH_3_ groups attached to a phenyl ring at the *p*- and *m*-positions. A singlet signal present at δ_H_ 5.45 ppm showed two protons of the OCH_2_ group. The signals at δ_H_ 6.87 ppm and 6.89 ppm were ascribed to two methine protons. A multiplet signal appearing in between δ_H_ 7.28 ppm and 7.87 ppm confirmed nine aromatic protons.

In the ^13^C-NMR spectrum of **5h**, the signal at δ_C_ 11.39 ppm indicated aliphatic CH_3_ carbon. Two signals appearing at δ_C_ 58.11 and 58.39 ppm confirmed two carbons of two methoxy groups attached to a phenyl ring and another signal at 69.46 ppm confirmed carbon of a OCH_2_ group attached to a triazole ring. Twelve signals appearing in between 110.24 and 153.13 ppm showed twelve aromatic carbons and a signal at 166.24 ppm explained the presence of the carbonyl carbon of an ester group of **5h**.

The distinguishing peak observed at m/z 451 in the ESI (Electrospray Ionization) mass spectrum confirmed the protonated molecular ion [M + H]^+^ of compound **5h**. A single crystal XRD study was not carried out as suitable crystals were not obtained from the crystallization of any of the synthesized compound. Various methods and solvents were tried for crystallization to get a suitable crystal for a single-crystal XRD study. However, all efforts resulted in an amorphous powder, which was not suitable for single-crystal XRD analysis.

### 2.2. Antimicrobial Activity

In the present study, the antimicrobial activities of synthesized compounds were screened against ten bacteria and two fungi using an in vitro well method [19,20]. The results are summarized below (Table 3 and Table 4). In particular, compounds **5a**, **5g**, **5h**, **5j**, and **5k** showed promising activity against tested bacteria at a 1 mg/mL concentration. Compound **5g** exhibited significantly potent antimicrobial activity against the tested bacteria. The MIC (Minimum Inhibitory Concentration) value of compound **5g** was found to be 31.25 µg/mL against *K. pneumonia*, *S. flexneri*, *P. vulgaris*, *S. aureus*-MRSA and *S. epidermidis.*

### 2.3. Anticancer Results

**Figure 3:**
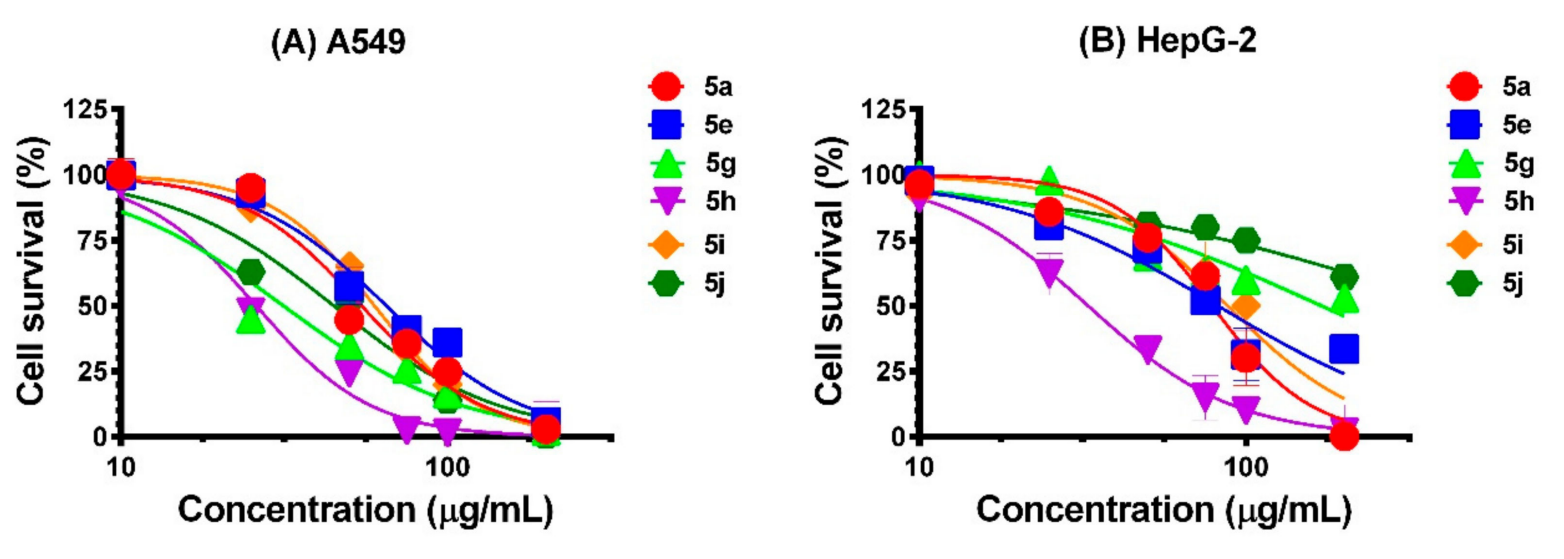
Effect of synthesized compounds against (**A**) A549 and (**B**) HepG-2 cells. Data were calculated using three independent experiments with mean ± SD.

Anticancer activity studies were performed for the synthesized compounds **5a**, **5e**, and **5g**–**j** (which showed higher antimicrobial activity than the others) against A549 and HepG-2 cells [15,21]. All the tested compounds showed good cytotoxic activity against A549 cells. However, **5a, 5e, 5i**, and **5j** showed good in vitro cytotoxic activity against both A549 and HepG-2 cells (Figure 3). However, **5a**, **5e**, and **5i** showed moderate activity against HepG-2 cells (Table 5). The cytotoxicity properties of synthesized compounds against A549 cells were observed at concentrations of 275 μM to 58 μM. The cytotoxic results showed that among all the tested compounds, **5a**, **5e**, **5g**, **5h**, **5i**, and **5j** showed promising anticancer activity against A549 cells. Compounds **5g** and **5h** showed potent cytotoxic activity against A549 cells with IC_50_ values of 72 and 58 µM, respectively (Table 5). A toxicity study of all synthesized compounds including **5a**, **5e**, and **5g**–**j** against human normal IMR90 cells showed no toxicity up to 250 µM (*vide infra*, supporting information).

### 2.4. Molecular Docking Studies

The synthesized compounds were subjected to molecular docking studies [15] with the DNA topoisomerase IV [21,22,23] and anaplastic lymphoma kinase [24] receptors in order to rationalize the biological studies. The docking study of the synthesized compounds was performed using AutoDock Tools (ADT) version 1.5.6 and AutoDock version 4.2.5.1 docking program [25,26] with the crystal structure of DNA topoisomerase IV (PDB ID: 4EMV) and anaplastic lymphoma kinase (PDB ID: 2XP2) [27]. The conformations of the docked ligands with receptors were evaluated in terms of energy, hydrogen bonding, polar, and hydrophobic interactions. The free energy of binding (FEB) was estimated for all the synthesized compounds **5a**–**l** and listed in Table 6.

**Figure 4:**
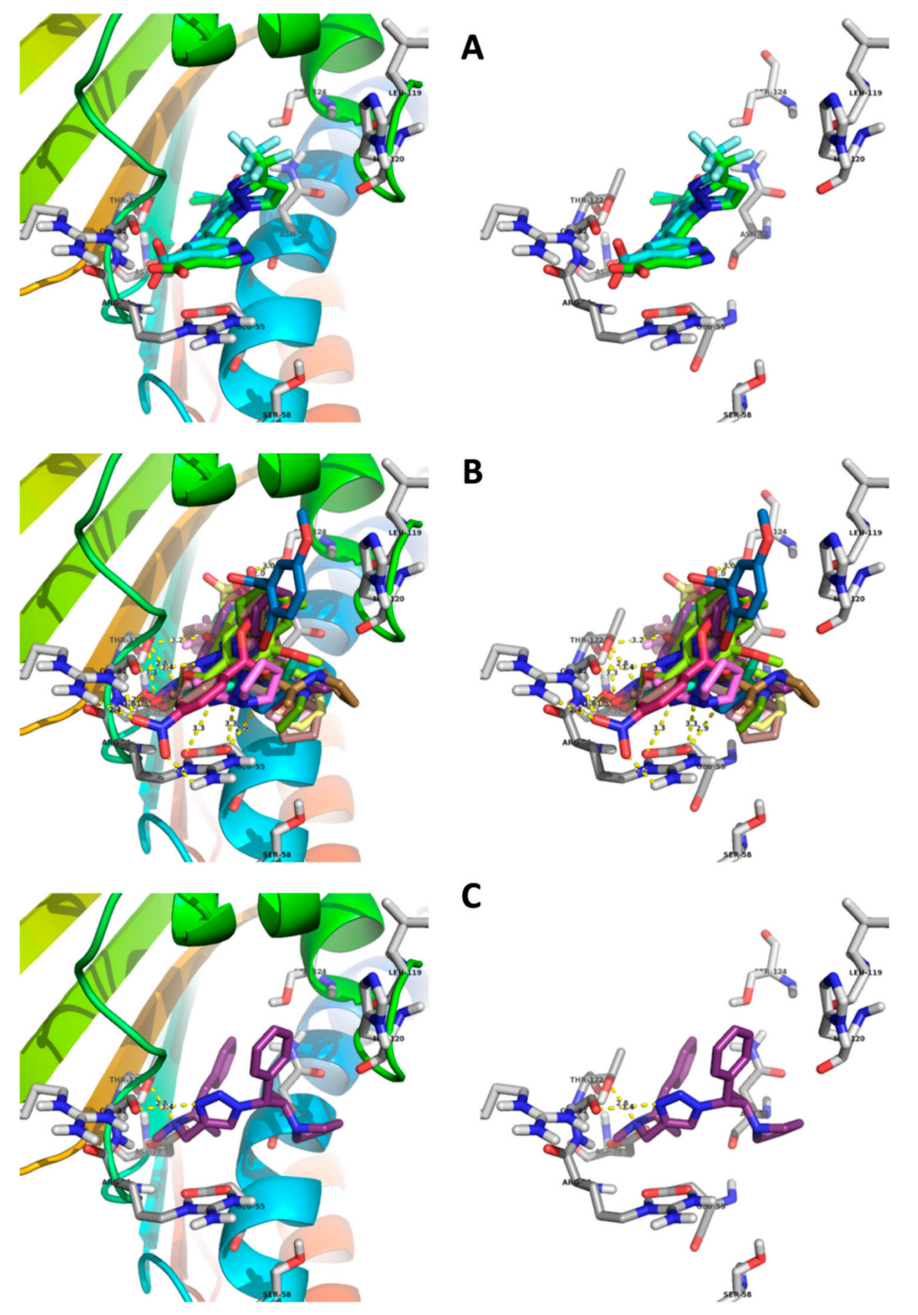
Molecular docking with 4EMV receptor: (**A**) method validation using a crystallized and docked ligand, (**B**) docking mode of all the compounds, and (**C**) docking mode of **5f**.

The molecular docking experiment established that the synthesized compounds **5a**–**l** had a good free energy of binding with the 4EMV receptor. These results revealed that the compounds **5a**–**l** exhibit free energy of binding values from −7.24 to −8.98 kcal/mol. Interestingly, compound **5f** exhibited good binding with the 4EMV receptor with a binding energy of −8.93 kcal/mol. In **5f**, N-CH_3_ formed a hydrogen bond with the H–O of THR-172 with the bond length of 2.8 Å. In addition, nitrogens of triazole interacted with the GLY-82 and formed the polar interactions. Furthermore, compound **5f** exhibited a hydrophobic interaction with ASN-51, GLU-55, GLY-82, HIS-120, and THR-172 amino acids. Binding interaction of the compound **5f** with the 4EMV receptor is shown in Figure 4.

**Figure 5:**
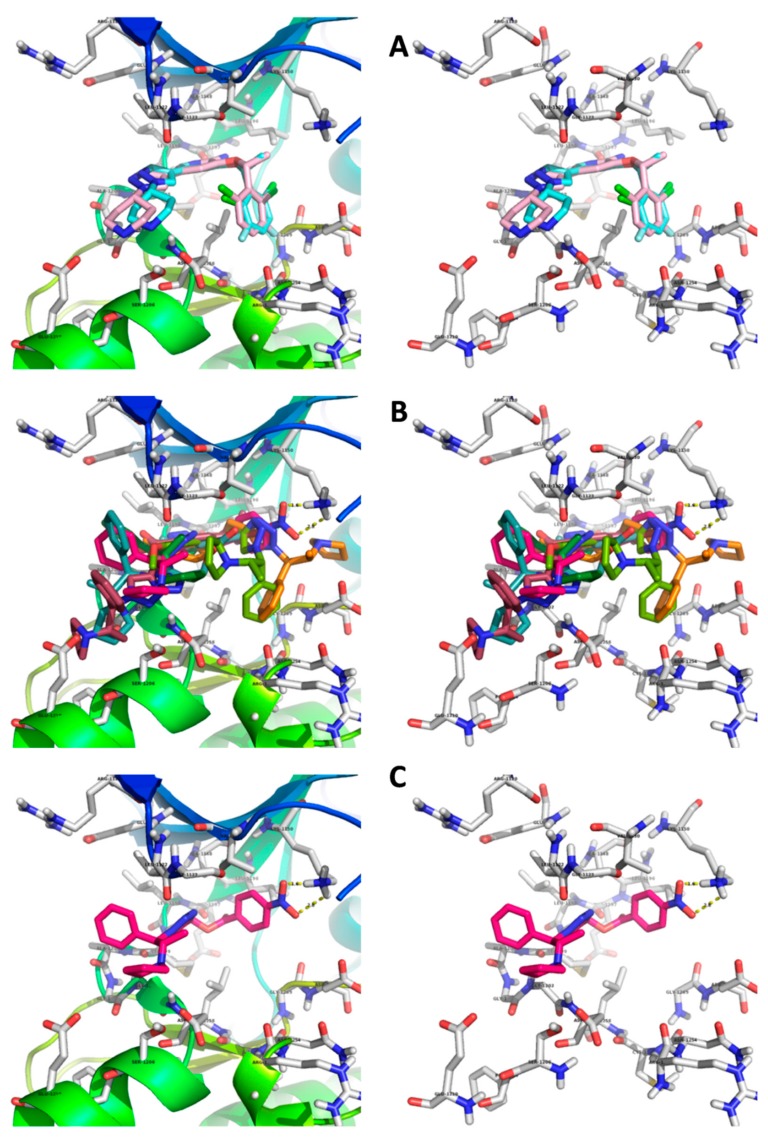
Molecular docking with the 2XP2 receptor: (**A**) method validation using a crystallized and docked ligand, (**B**) docking mode of all the compounds, and (**C**) docking mode of **5g.**

Docking experiments of synthesized compounds with the 2XP2 (ALK tyrosine kinase receptor) receptor revealed that all the docked compounds bind with the receptor and exhibits free energy of binding value from −6.55 to −8.72 kcal/mol. Compounds **5a**–**l** interacted with the active site amino acids of 2XP2 namely ARG-1120, LEU-1122, GLY-1123, VAL-1130, GLU-1132, ALA-1148, LYS-1150, LEU-1196, GLU-1197, LEU-1198, MET-1199, ALA-1200, GLY-1201, GLY-1202, ASP-1203, SER-1206, PHE-1207, GLU-1210, ARG-1253, ASN-1254, CYS-1255, LEU-1256, GLY-1269, and ASP-1270. Compound **5g** exhibited better binding with the 2XP2 receptor, displaying a binding energy of −8.12 kcal/mol. In compound **5g**, two oxygens of NO_2_ interacted with the N–H of LYS-1150 and formed two hydrogen bonds with the bond lengths of 1.6 Å and 2.8 Å. In addition, C=O formed a polar interaction with the GLU-1197 amino acid. Furthermore, compound **5g** exhibited a hydrophobic interaction with the LEU-1122, ALA-1148, LYS-1150, LEU-1196, LEU-1198, ALA-1200, GLY-1201, GLY-1202, and LEU-1256 amino acids. The binding interaction of the compound **5g** with the 2XP2 receptor is shown in Figure 5.

## 3. Materials and Methods

*Synthesis of 1-((1R,2R)-1-chloro-1-phenylpropan-2-yl)pyrrolidine hydrochloride***2**: Thionyl chloride (49.3 g, 0.41 mol) was slowly added to the mixture of pyrrolidinylnorephedrine hydrochloride (50 g, 0.21 mol) and chloroform (200 mL) at 25–30 °C. The mass was heated to 60–65 °C and refluxed for 3 h. Progress of the reaction was monitored using TLC (Thin Layer Chromatography) (mobile phase: 100% dichloromethane). After completion of the reaction, chloroform and unreacted thionyl chloride were distilled out completely. The mass was stirred with 200 mL of acetone for 30 min, and the obtained solid was filtered and dried under vacuum at 65 °C; a white crystalline solid was produced, weight 38g (70%).

*Synthesis of 1-((1S,2R)-1-azido-1-phenylpropan-2-yl)pyrrolidine***3**: Compound **2** (25 g, 0.096 mol) was added in lots to the solution of sodium azide (9.4 g, 0.14 mol, 1.5 mol equiv.) in dimethylformamide (100 mL) at 20–25 °C. The contents were stirred at 20–25 °C for 12 h. Progress of reaction was monitored via watching the formation of sodium chloride as a white precipitate during the stirring. Progress of the reaction was further monitored using TLC (mobile phase: 100% dichloromethane). After completion of the reaction, the mass was poured into ice water (300 mL) and basified with sodium hydroxide (pH about 10). Ethyl acetate (100 mL) was added, stirred for 10 min, and the organic layer was separated and dried over sodium sulfate. Ethyl acetate was distilled off completely using a rotavap at 60 °C to get the azide **3** as a syrupy mass and it was preserved in a refrigerator. It was used as such in the next step without any further purification. Weight of the azide **3**: 15 g (68%).

*Preparation of catalyst CuI/Al_2_O_3_:* Neutral alumina (100g) and copper iodide (10g) and methanol (300 mL) were mixed and heated for 1.0 h at 55–60 °C. Methanol was distilled off completely and the free-flowing white powder was dried at 75 °C for 6 h. The obtained catalyst was stored under nitrogen.

### 3.1. Pharmacological Activities

Antimicrobial activity was carried out as described previously [19,20]. Briefly, 20 mL of sterile Mueller Hinton agar (MHA) was poured in the petri plates and kept for solidification. After solidification, the test cultures were swabbed on the top of the media and kept for 10 min to dry. The required concentration (1 mg/well) of synthesized compounds **5a**–**l** was added to each well and left for 30 min at RT for compound diffusion. Ketoconazole (fungi) and streptomycin (bacteria) used for positive controls. The zone of inhibition was recorded in mm and the experiment was repeated twice.

### 3.2. Minimum Inhibitory Concentration (MIC)

The MIC experiment was carried out according to the standard reference methods [19,20]. Briefly, the required concentrations of synthesized compounds **5a**–**l** were dissolved in DMSO (Dimethylsulfoxide) in concentrations of 500 µg/mL, 250 µg/mL, 125 µg/mL, 62.5 µg/mL, 31.25 µg/mL, 15.6 µg/mL, and 0.78 µg/mL. An inoculum of 100 µL from each well (96-well plate) was inoculated. For the positive control, ketoconazole (fungi) and streptomycin (bacteria) was used. The MIC values for the tested bacteria were identified as the lowest inhibiting concentration of compound on the agar plate in visual growth.

### 3.3. Cytotoxicity Properties 

The cytotoxic study was carried out as reported previously [15,21]. Briefly, IMR90, HepG-2, and A549 cells were grown in DMEM (Dulbecco’s Modified Eagle Medium) that contained 10% fetal bovine serum and 1% antibiotics. A total of 15 × 10^3^ cells/well were seeded in 96-well plates and incubated under humidified conditions. Cells were treated with varying concentrations of synthesized triazoles for 24 h. After a 24 h treatment, 20 µL aqueous one solution reagent was added to each well and incubated for 3–4 h under a humidified condition. For IMR90 cells, MTT (MTT = 3-(4,5-dimethyl-2-thiazolyl)-2,5-diphenyl-2H-tetrazolium bromide) (5 mg/mL) was added and incubated for 3–4 h in the dark. After incubation, 100 µL of a 10% SDS (Sodium Dodecyl Sulfate) solution (0.01% HCl) was added to each well. The data was taken using an ELISA (enzyme-linked immunosorbent assay) reader at the absorbance of 490 nm for HepG-2 and A549 cells and 570 nm for IMR90 cells. The cell death was calculated using the following formula: Inhibition (%) = ((A − B)/A) × 100 (A—control group, B—treated group).

### 3.4. General Procedure for the Synthesis of β-pyrrolidino-1,2,3-triazoles ***5a–l***: 

Propargyl alcohol **4a** (0.53g, 9.5 mmol) was added to a solution of azide **3** (2.0g, 8.6 mmol) taken in a mixture of solvents (30 mL—MeOH/THF (Tetrahydrofuran)/water in equal amounts) followed by diisopropylethylamine (1.35 g, 10.4 mmol) and catalyst (CuI/Al_2_O_3_ 5 mol%) at 20–25 °C for 8–10 h. Completion of the reaction was monitored using TLC (mobile phase: 10% methanol in dichloromethane). After completion of the reaction, the catalyst was removed via filtration and preserved for recycling. The filtrate was concentrated in a rotavap to remove the solvents. Ethyl acetate (50 mL) was added to the concentrated mass and the organic layer was separated. The organic layer was washed with water (20 mL), dried over sodium sulfate, and completely concentrated to produce a semi-solid. The semi-solid was triturated with acetone (5 mL), filtered, and dried at 60 °C under vacuum. This produced compound **5a** as a brown solid, weight 2.36g (95%). The method as mentioned above was used to make compound **5b**–**l** using terminal alkynes **4b**-**l** instead of **4a**.

(1*R*,2*S*)-*1-(1-(1-Phenyl-2-(pyrrolidin-1-yl)propyl)-1H-1,2,3-triazol-4-yl)methanol (***5a***)*

Pale brown solid, yield 91%, MP: 160–161 °C, IR (KBr, cm^−1^): 3252, 1354, 1009, 905, and 760; ^1^H-NMR (500 MHz, CDCl_3_); δ_H_ (ppm) 0.94 (3H, d, *J* = 6.6 Hz, CH_3_), 1.63 (4H, m, C–CH_2_–CH_2_–C), 2.53–2.63 (4H, m, CH_2_–N–CH_2_), 3.88–3.92 (1H, m, N–CH–CH–CH_3_), 4.76 (2H, s, CH_2_–O), 5.58 (1H, d, *J* = 10.0 Hz, CH–CH–CH_3_), 7.27–7.70 (6H, m, aromatic); ^13^C-NMR (125 MHz, DMSO-*d*_6_); δ_C_ (ppm) 11.65 (CH_3_), 23.68 (C_3_ & C_4_ carbons of pyrrolidinyl ring), 48.19 (C_2_ & C_5_ carbons of pyrrolidinyl ring), 56.7 (CH–CH–CH_3_), 58.50 (CH–CH–CH_3_), 69.32 (OCH_2_), 121.46, 127.75, 128.55, 128.93, 137.80, and 148.19; ESI-MS: *m/z* 287 ([M + H]^+^, 100%); Anal. Calcd for C_16_H_22_N_4_O: C, 67.11; H, 7.74; N, 19.56 Found C, 67.24; H, 7.86; N, 19.68.

(1*R*,2*S*)-*1-((1-(1-Phenyl-2-(pyrrolidin-1-yl)propyl)-1H-1,2,3-triazol-4-yl)methyl) cyclohexanol, (***5b***)*

Brown solid, yield 84%, MP: 137–138 °C, IR (KBr, cm^−1^): 3375, 1350, 1001, 982, and 748; ^1^H-NMR (500 MHz, CDCl_3_); δ_H_ (ppm) 1.00(3H, d, *J* = 6.6 Hz, CH_3_), 1.18–1.32 (2H, m, five CH_2_ of cycloxyl ring at C_4_), 1.35–1.52 (4H, m, two CH_2_′s of cycloxyl ring at C_4_ & C_4_), 1.66–1.84 (4H, m, two CH_2_′s of pyrrolidinyl ring at C_3_ & C_4_), 1.86 (2H, s, CH_2_ attached to triazole ring), 1.98–2.21 (4H, m, two CH_2_′s of pyrrolidinyl ring at C_2_ & C_5_), 2.32 (1H, m, CH–CH_3_), 2.54–2.63 (4H, m, two CH_2_′s of cycloxyl ring at C_2_ & C_6_), 3.93 (1H, s, OH), 5.64 (1H, m, CH–CH–CH_3_), 7.23–7.59 (6H, m, aromatic); ^13^C-NMR (125 MHz, CDCl_3_); δ_C_ (ppm) 11.23 (CH_3_), 22.11 (C_4_ carbon of cyclohexyl ring), 23.58 (C_3_ & C_4_ carbon of pyrrolidinyl ring), 25.41 & 25.93 (C_3_ & C_5_ carbons of cyclohexyl ring), 26.77 (C_2_ & C_6_ carbons of cyclohexyl ring), 48.26 (C_2_ & C_5_ carbons of pyrrolidinyl ring), 49.71 (CH–CH_3_), 51.22 (CH–CH–CH_3_), 66.50 (C_1_ carbon of cyclohexyl ring), 120.48, 124.46, 127.66, 128.69, 135.46, and 148.91; ESI-MS: *m/z* 369 ([M + H]^+^, 100%); Anal. Calcd for C_22_H_32_N_4_O: C, 71.70; H, 8.75; N, 15.20; Found C, 71.81; H, 8.87; N, 15.32.

(1*R*,2*S*)- *(1-(1-Phenyl-2-(pyrrolidin-1-yl)propyl)-1H-1,2,3-triazol-4-yl)methyl cinnamate, (***5c***)*

Brownish yellow solid, yield 88%, MP: 140–141 °C, IR (KBr, cm^−1^): 1713, 1599, 1578, 1369, 1275, 1011, 978, 847, and 775; ^1^H-NMR (500 MHz, CDCl_3_); δ_H_ (ppm) 0.95 (3H, d, *J* = 6.5 Hz, CH_3_), 1.77–2.01 (4H, m, two CH_2_′s of pyrrolidinyl ring at C_3_ & C_4_), 2.48–2.59 (4H, m, two CH_2_′s of pyrrolidinyl ring at C_2_ & C_5_), 3.81–3.87 (2H, m, OCH_2_), 5.52–5.54 (1H, m, N–CH–CH_3_), 6.45 (1H, d, *J* = 16 Hz, CH–CH–CH_3_), 7.28–7.65 (12H, m, aromatic); ^13^C-NMR (100 MHz, CDCl_3_); δ_C_ (ppm) 11.91 (CH_3_), 22.14 (C_3_ & C_4_ carbon of pyrrolidinyl ring), 48.12 (C_2_ & C_5_ carbons of pyrrolidinyl ring), 48.45 (CH–CH_3_), 58.65 (CH–CH–CH_3_), 69.55 (O–CH_2_), 112.04, 116.30, 119.29, 124.25, 127.84, 128.99, 132.25, 135.02, 141.83, 158.54, and 169.30 (ester carbonyl); ESI-MS: *m/z* 417 ([M + H]^+^, 100%); Anal. Calcd for C_25_H_28_N_4_O_2_: C, 72.09; H, 6.78; N, 13.45 Found C, 72.18; H, 6.86; N, 13.52.

(1*R*,2*S*)- *(1-(1-Phenyl-2-(pyrrolidin-1-yl)propyl)-1H-1,2,3-triazol-4-yl)methyl benzoate, (***5d***)*

Pale brown solid, yield 75%, MP: 129–130 °C, IR (KBr, cm^−1^): 1730, 1589, 1568, 1331, 1286, 1018, 947, 852, and 773; ^1^H-NMR (500 MHz, CDCl_3_); δ_H_ (ppm) 0.92 (3H, d, *J* = 3.0Hz, CH_3_), 1.58–1.66 (4H, m, two CH_2_′s of pyrrolidinyl ring at C_3_ & C_4_), 2.51–2.59 (4H, m, two CH_2_′s of pyrrolidinyl ring at C_2_ & C_5_), 5.47 (2H, s, OCH_2_), 5.54–5.56 (1H, m, N–CH–CH_3_), 6.86–6.68 (1H, d of d, *J* = 8.8 & 3.1 Hz, CH–CH–CH_3_), 7.27–7.89 (11H, m, aromatic); ^13^C-NMR (500 MHz, CDCl_3_); δ_C_ (ppm) 11.93 (CH_3_), 23.60 (C_3_ & C_4_ carbon of pyrrolidinyl ring), 48.45 (C_2_ & C_5_ carbons of pyrrolidinyl ring), 55.68 (CH–CH_3_), 58.91 (CH–CH–CH_3_), 69.09 (O–CH_2_), 112.02, 116.25, 119.29, 124.25, 127.84, 128.68, 128.99, 132.25, 137.46, 141.84, 158.68, and 166.80 (ester carbonyl); ESI-MS: *m/z* 391([M + H]^+^, 100%); Anal. Calcd for C_23_H_26_N_4_O_2_: C, 70.75; H, 6.71; N, 14.35. Found C, 70.86; H, 6.80; N, 14.47.

(1*R*,2*S*)- *(1-(1-Phenyl-2-(pyrrolidin-1-yl)propyl)-1H-1,2,3-triazol-4-yl)methyl-2-bromo-5-methoxy benzoate, (***5e***)*

Pale brown solid, yield 92%, MP: 111–112 °C, IR (KBr, cm^−1^): 1725, 1589, 1570, 1315, 1286, 1018, 947, and 852; ^1^H-NMR (400 MHz, DMSO-*d*_6_); δ_H_ (ppm) 0.99–1.04 (3H, m, CH_3_), 1.57–1.80 (4H, m, two CH_2_′s of pyrrolidinyl ring at C_3_ & C_4_), 2.56–2.75 (4H, m, two CH_2_′s of pyrrolidinyl ring at C_2_ & C_5_), 3.80 (3H, s, OCH_3_) 5.48 (2H, s, OCH_2_), 5.78 (1H, m, N–CH–CH_3_), 6.88–6.91 (1H, dd, *J* = 8.8 & 3.1 Hz, CH–CH–CH_3_), 7.28–7.94 (9H, m, aromatic); ^13^C-NMR (100MHz, DMSO-*d*_6_); δ_C_ (ppm) 11.31 (CH_3_), 23.70 (CH-CH-CH_3_), 47.94 (C_3_ & C_4_ carbons of pyrrolidinyl ring), 55.69 (C_2_ & C_5_ carbons of pyrrolidinyl ring), 58.57 (CH–CH–CH_3_), 58.96 (OCH_3_), 69.49 (OCH_2_), 112.06, 116.29, 119.31, 124.00, 127.81, 128.57, 128.93, 132.31, 135.01, 137.67, 141.65, 158.57, and 165.83 (ester carbonyl); ESI-MS: *m/z* 499 and 501 ([M + H]^+^, 100%), in the ratio of 1:1); Anal. Calcd for C_24_H_27_BrN_4_O_3_: C, 57.72; H, 5.45; N, 11.22 Found C, 57.84; H, 5.52; N, 11.29.

(1*R*,2*S*)-*N-Methyl-1-phenyl-N-((1-(1-phenyl-2-(pyrrolidin-1-yl)propyl)-1H-1,2,3-triazol-4-yl)methyl)propan-2-amine, (***5f***)*

Brown solid, yield 75%, MP: 118–119 °C, IR (KBr, cm^−1^): 1551, 1495, 1366, 1258, 1026, 959, 849, and 741; ^1^H-NMR (400 MHz, DMSO-*d*_6_); δ_H_ (ppm) 1.03 (3H, d, *J* = 6.4 Hz, CH_3_), 1.26 (3H, m, CH_3_), 1.54–1.59 (4H, m, two CH_2_′s of pyrrolidinyl ring at C_3_ & C_4_), 2.29–2.30 (4H, m, two CH_2_′s of pyrrolidinyl ring at C_2_ & C_5_), 2.43–2.48 (2H, m), 2.56–2.58 (2H, d, *J* = 7.0 Hz. CH–CH_2_), 2.95–2.97 (2H, m), 3.82 (3H, s, N-CH_3_), 5.45 (1H, d, *J* = 9.8 Hz, CH–CH–CH_3_), 7.08–7.47 (11H, m, aromatic); ^13^C-NMR (100 MHz, DMSO-*d*_6_); δ_C_ (ppm) 10.92 & 11.37 (CH_2_–CH–CH_3_ & CH–CH–CH_3_), 30.28 (CH_2_–Ph), 36.02 (CH–CH–CH_3_), 38.41 (CH_2_–CH–CH_3_), 47.95 (C_3_ & C_4_ carbons of pyrrolidinyl ring), 48.97 (C_2_ & C_5_ carbons of pyrrolidinyl ring), 58.55 (CH–CH–CH_3_), 59.57 (N–CH_2_), 115.27, 115.99, 124.06, 126.29, 127.94 128.38, 129.90, 137.59, 142.27, and 159.02; ESI-MS: *m/z* 418 ([M + H]^+^, 100%); Anal. Calcd for C_26_H_35_N_5_: C, 74.78; H, 8.45; N, 16.77 Found C, 74.92; H, 8.52; N, 16.83.

(1*R*,2*S*)- *(1-(1-Phenyl-2-(pyrrolidin-1-yl)propyl)-1H-1,2,3-triazol-4-yl)methyl 4-nitrobenzoate, (***5g***)*

Yellowish brown powder, yield 69%, MP: 137–138 °C, IR (KBr, cm^−1^): 1722, 1607, 1528, 1348, 1271, 1015, 937, 854, and 787; ^1^H-NMR(400MHz, DMSO-*d*_6_); δ_H_ (ppm) 1.01(3H, d, *J* = 5.9 Hz, CH_3_), 1.58–1.71 (4H, m, two CH_2_′s of pyrrolidinyl ring at C_3_ & C_4_), 2.57–2.67 (4H, m, two CH_2_′s of pyrrolidinyl ring at C_2_ & C_5_), 4.00 (1H, m, CH–CH_3_), 5.52 (2H, s, OCH_2_), 5.71 (1H, brs, CH–CH–CH_3_), 7.28–8.29 (10H, m, aromatic); ^13^C-NMR (100 MHz, DMSO-*d*_6_); δ_C_ (ppm) 11.19 (CH_3_), 23.69 (N–CH_2_–CH_2_), 47.76 (N–CH_2_–CH_2_), 58.30 (N–CH–CH_3_), 59.03 (N–CH–Ph), 69.96 (OCH_2_), 123.50, 124.01, 127.888, 128.62, 128.94, 130.85, 135.30, 137.57, 141.16, 150.63, and 164.57 (carbon of ester carbonyl); ESI-MS: *m/z* 436 ([M + H]^+^, 100%); Anal. Calcd for C_23_H_25_N_5_O_4_: C, 63.44; H, 5.79; N, 16.08 Found C, 63.55; H, 5.82; N, 16.14.

(1*R*,2*S*)- *(1-(1-Phenyl-2-(pyrrolidin-1-yl)propyl)-1H-1,2,3-triazol-4-yl)methyl-3,4-dimethoxy-benzoate, (***5h***)*

Brown powder, yield 86%, MP 108–109 °C, IR (KBr, cm^−1^): 1701, 1599, 1514, 1385, 1271, 1020, 949, and 843; ^1^H-NMR (400MHz, DMSO-*d*_6_); δ_H_ (ppm) 1.02 (3H, d, *J* = 1.2 Hz, CH_3_), 1.66 (4H, m, two CH_2_′s of pyrrolidinyl ring at C_3_ & C_4_), 2.59–2.61 (4H, m, two CH_2_′s of pyrrolidinyl ring at C_2_ & C_5_), 3.92 (3H, s, *p*-OCH_3_), 3.94 (3H, s, *m*-OCH_3_), 5.45 (2H, s, OCH_2_), 6.87 (1H, m), 6.89 (1H, d, *J* = 8.5 Hz), 7.28–7.87 (9H, m, aromatic); ^13^C-NMR (100 MHz, DMSO-*d*_6_); δ_C_ (ppm) 11.39 (CH_3_), 13.85 (N–CH_2_–CH_2_), 23.64 (N–CH_2_–CH_2_), 47.86 (N–CH–CH_3_), 55.99 (N–CH–Ph), 58.11 (*p*-OCH_3_), 58.39 (*m*-OCH_3_), 69.96 (OCH_2_), 110.24, 112.05, 122.38, 123.84, 123.93, 127.88, 128.53, 128.89, 137.65, 142.14, 148.58, 153.13, and 166.24 (carbon of ester carbonyl); ESI-MS: *m/z* 451 ([M + H]^+^, 100%); Anal. Calcd for C_25_H_30_N_4_O_4_: C, 66.65; H, 6.71; N, 12.44 Found C, 66.74; H, 6.83; N, 12.54.

(1*R*,2*S*)- *(1-(1-Phenyl-2-(pyrrolidin-1-yl)propyl)-1H-1,2,3-triazol-4-yl)methyl 4-tert-butyl benzoate, (***5i***)*

Brown solid, yield 90%, MP: 145–147 °C, IR (KBr, cm^−1^): 1719, 1495, 1393, 1273, 1016, 951, 852, and 775; ^1^H-NMR (400 MHz, DMSO-*d*_6_); δ_H_ (ppm) 0.93 (3H, d, *J* = 16.3 Hz, CH_3_), 1.56 (9H, s, tert-butyl), 2.37 (4H, m, two CH_2_′s of pyrrolidinyl ring at C_3_ & C_4_), 2.49–2.56 (m, 4H two CH_2_′s of pyrrolidinyl ring at C_2_ & C_5_), 3.83–3.86 (1H, m, CH–CH_3_), 5.42 (2H, s, OCH_2_), 5.52 (1H, m, N–CH–Ph), 7.28–7.84 (10H, m, aromatic); ^13^C-NMR (100 MHz, DMSO-*d*_6_); δ_C_ (ppm) 11.59 (CH_3_), 21.23, 23.61 (N–CH_2_–CH_2_), 31.09 (C(CH_3_)_3_), 48.09 (N–CH_2_–CH_2_), 58.23 (N–CH–Ph), 58.60, 69.39, 123.98, 125.36, 126.87, 128.24, 128.60, 129.79, 130.25, 136.13, 138.14, 142.16, 166.65, and 166.24 (carbon of ester carbonyl); ESI-MS: *m/z* 447 ([M + H]^+^, 100%);Anal. Calcd for C_27_H_34_N_4_O_2_: C, 72.62; H, 7.67; N, 12.55 Found C, 72.74; H, 7.78; N, 12.62.

(1*R*,2*S*)- *(1-(1-Phenyl-2-(pyrrolidin-1-yl)propyl)-1H-1,2,3-triazol-4-yl)methyl 3-methylbenzoate, (***5j***)*

Pale brown solid, yield 84%, MP: 150–152 °C, IR (KBr, cm^−1^): 1707, 1589, 1389, 1277, 1053, 953, 859, and 770; ^1^H-NMR (400 MHz, DMSO-*d*_6_); δ_H_ (ppm) 0.93 (3H, d, *J* = 6.2 Hz, CH_3_), 1.56 (4H, m, two CH_2_’s of pyrrolidinyl ring at C_3_ & C_4_), 2.37 (3H, s, Ph-CH_3_) 2.49–2.56 (m, 4H, two CH_2_′s of pyrrolidinyl ring at C_2_ & C_5_), 3.83–3.86 (1H, m, CH–CH_3_), 5.45 (2H, s, OCH_2_), 5.48–5.52 (1H, m, N–CH–Ph), 7.27–7.84 (10H, m, aromatic); ^13^C-NMR (100 MHz, DMSO-*d*_6_); δ_C_ (ppm) 11.59 (CH_3_), 21.23 (C_3_ & C_4_ carbon of pyrrolidinyl ring), 23.61 (CH_3_ attached to phenyl ring), 48.09 (C_2_ & C_5_ carbons of pyrrolidinyl ring), 58.23 (CH–CH_3_), 58.6 (CH–CH–CH_3_), 69.39 (O–CH_2_), 123.33, 126.87, 127.24, 128.60, 129.79, 133.88, 137.56, 138.14, 142.16, and 166.65 (ester carbonyl); ESI-MS: *m*/*z* 405 (M + H, 100%); Anal. Calcd for C_24_H_28_N_4_O_2_: C, 71.26; H, 6.98; N, 13.85 Found C, 71.38; H, 7.07; N, 13.97.

(1*R*,2*S*)- *(1-(1-Phenyl-2-(pyrrolidin-1-yl)propyl)-1H-1,2,3-triazol-4-yl)methyl 2-chlorobenzoate**,** (***5k***)*

Brownish yellow solid, yield 79%, MP: 101–102 °C, IR (KBr, cm^−1^): 1719, 1589, 1391, 1265, 1049, 943, 851, and 768; ^1^H-NMR (400 MHz, DMSO-*d*_6_); δ_H_ (ppm) 0.98 (3H, d, *J* = 6.2 Hz, CH_3_), 1.53 (4H, m, two CH_2_′s of pyrrolidinyl ring at C_3_ & C_4_), 2.56 (m, 4H, two CH_2_′s of pyrrolidinyl ring at C_2_ & C_5_), 3.94 (1H, m, CH–CH_3_), 5.47 (2H, s, OCH_2_), 5.64 (1H, m, N–CH–Ph), 7.28–7.86 (10H, m, aromatic); ^13^C-NMR (100 MHz, DMSO-*d*_6_); δ_C_ (ppm) 11.81 (CH_3_), 21.23 (C_3_ & C_4_ carbon of pyrrolidinyl ring), 48.42 (C_2_ & C_5_ carbons of pyrrolidinyl ring), 49.82 (CH–CH_3_), 59.12 (CH–CH–CH_3_), 71.98 (O–CH_2_), 122.24, 123.91, 124.11, 126.34, 128.44, 129.06, 130.36, 135.66, 136.15, 137.23, 138.23, 149.10, and 166.23 (ester carbonyl); ESI-MS: [M + H]^+^ at *m/z* 425 and 427 in the ratio of 3:1; Anal. Calcd for C_23_H_25_ClN_4_O_2_: C, 65.01, H, 5.93; N, 13.19 Found C, 65.13; H, 5.98; N, 13.27.

(1*R*,2*S*)-*((1-(1-Phenyl-2-(pyrrolidin-1-yl)propyl)-1H-1,2,3-triazol-4-yl)methyl 2-phenylbutanoate, (***5l***)*

Brown solid, yield 70%, MP: 127–129 °C, IR (KBr, cm^−1^): 1738, 1601, 1381, 1032, 997, 851, and 770; ^1^H-NMR (400 MHz, DMSO-*d*_6_); *δ*_H_ (ppm) 0.82 (3H, t, *J* = 7.3 Hz, CH_2_–CH_3_), 0.98 (3H, t, *J* = 6.4 Hz, CH_3_), 1.60–163 (4H, m, two CH_2_′s of pyrrolidinyl ring at C_3_ & C_4_), 1.76–1.78 (1H, m), 2.04–2.06 (1H, m), 2.50–2.67 (4H, m), 3.44–3.47 (1H, m, CH–CH_3_), 3.84 (m, 1H), 5.16–5.24 (m, 2H, OCH_2_), 5.56 (1H, m), 7.26–7.59 (11H, m, aromatic); ^13^C-NMR (100 MHz, DMSO-*d*_6_); δ_C_ (ppm) 12.07 (CH_2_–CH_3_), 18.12 (CH_3_), 23.55 (CH_2_–CH_3_), 26.66 (C_3_ & C_4_ carbon of pyrrolidinyl ring), 48.69 (C_2_ & C_5_ carbons of pyrrolidinyl ring), 53.22 (CH–CH_3_), 58.02 ((C=O)–CH–Ph), 59.11 (CH–CH–CH_3_), 68.79 (O–CH_2_), 123.59, 127.23, 127.77, 127.96, 128.55, 128.68, 128.97, 137.43, 138.77, 142.48, and 173.88 (ester carbonyl); ESI-MS: *m/z* 433 [M + H]^+^, 100%; Anal. Calcd for C_26_H_32_N_4_O_2_: C, 72.19; H, 7.46; N, 12.95 Found C, 72.33; H, 7.54; N, 12.99.

Recovery and reuse of catalyst CuI/Al_2_O_3_: The recovered catalyst from the reaction was washed thoroughly with methanol and dried at 75 °C for 6 h and reused. During this study, we used the catalyst ten times with the same activity in terms of the progress of the reaction, which was generally completed in 16–20 h. The reaction time was increased to 20–30 h for the subsequent reactions using the recycled catalyst after 10 cycles.

## 4. Conclusions

In summary, copper-iodide-infused neutral alumina catalyst was developed and used for the efficient synthesis of 1,4-disubstituted-1,2,3-triazoles **5a**–**l** using a click chemistry approach from pyrrolidinylnorephedrine **1**. The advantages of the prepared catalyst over previous reports were its easy preparation, fast reaction times, and recyclability of the used catalyst. This effective heterogeneous catalyst was reused for ten cycles by isolating the desired product in high yields using simple filtration. The obtained compounds **5a**–**l** showed good antimicrobial activities when compared with standard drugs streptomycin and ketoconazole. Triazoles **5a**, **5e**, and **5g**–**j**, which had high antimicrobial activities were also screened for cytotoxicity activity and were found to have influential cytotoxicity activities against A549 and HepG-2 cell lines. Among these tested compounds, **5g** and **5h** showed potent cytotoxic activity. In order to rationalize the biological studies, the docking was done with the DNA topoisomerase IV and anaplastic lymphoma kinase receptors. The studies revealed that the synthesized compounds exhibited better binding energy with the targeted receptors.

## Figures and Tables

**Table 1:** Optimization of reaction conditions.

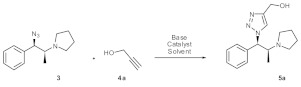						
Entry	Base (1.2 mol equiv.)	Catalyst (mol%)	Solvent	Temp (°C)	Time (h)	5a Yield (%)
1	DIPEA	CuI (10)	n-BuOH	25–30	10	75
2	DIPEA	CuBr (10)	n-BuOH	25-30	10	15
3	DIPEA	CuI (5)	n-BuOH	25–30	10	46
4	DIPEA	CuI (20)	n-BuOH	25–30	10	85
5	DIPEA	CuI (10)	H_2_O	25–30	10	48
6	DIPEA	CuI (10)	n-BuOH/H_2_O(1:1)	25–30	10	80
7	DIPEA	CuI (10)	EtOH	25–30	10	Trace
8	DIPEA	CuI (10)	EtOH	45–50	20	15
9	DIPEA	CuI (10)	EtOH/H_2_O(1:1)	45–50	20	27
10	DIPEA	CuI (10)	MeOH/THF/water(1:1:1)	25–30	10	82
11	DIPEA	10% CuI/Al_2_O_3_ (5)	MeOH/THF/water(1:1:1)	25–30	10	95
12	DIPEA	10% CuI/Al_2_O_3_ (10)	MeOH/THF/water(1:1:1)	25–30	10	92
13	DIPEA	10% CuI/Al_2_O_3_ (5)	EtOH/THF/water(1:1:1)	25	10	89
14	TEA	10% CuI/Al_2_O_3_ (10)	n-BuOH/water(1:1:1)	25	10	65
15	DIPEA	CuI (10)	THF	25–30	20	60
16	TEA	CuI (10)	n-BuOH	25–30	10	Trace
17	TEA	CuI (10)	THF	25–30	10	Trace
18	KOH	CuI (20)	n-BuOH	25–30	10	Trace
19	KOH	CuI (20)	n-BuOH	50–55	20	Trace

* Isolated yields after recrystallization in acetone.

**Table 2:** Synthesis of compounds **5a**–**l** from optimized conditions.

Entry	Terminal Alkyne	β-pyrrolidino-1,2,3-triazole	Yield%	Entry	Terminal Alkyne	β-pyrrolidino-1,2,3-triazole		Yield %
1	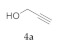 **4a**	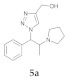 **5a**	95	7	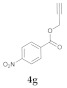 **4g**	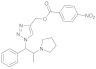 **5g**		80
2	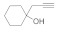 **4b**	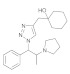 **5b**	84	8	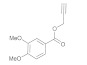 **4h**	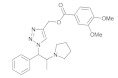 **5h**		86
3	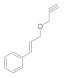 **4c**	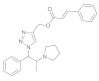 **5c**	88	9	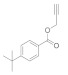 **4i**	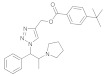 **5i**		90
4	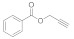 **4d**	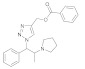 **5d**	96	10	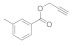 **4j**	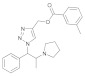 **5j**		84
5	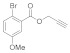 **4e**	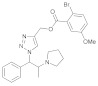 **5e**	92	11	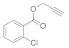 **4k**	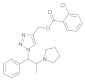 **5k**		79
6	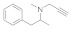 **4f**	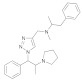 **5f**	82	12	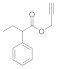 **4l**	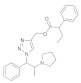 **5l**		70

**Table 3:** Antimicrobial activity of the synthesized compound using a well method (zone of inhibition in mm) (1 mg/well).

Organism	5a	5b	5c	5d	5e	5f	5g	5h	5i	5j	5k	5l	C
**Bacteria**													
*Enterobacter aerogenes*	14	12	14	10	10	-	22	17	14	-	22	-	22
*Staphylococcus aureus*	12	10	16	13	12	-	16	15	16	15	18	-	14
*Staphylococcus epidermidis*	14	10	15	13	10	17	28	16	11	14	-	-	26
*Staphylococcus aureus*-MRSA	13	12	18	16	10	15	24	20	14	14	17	-	30
*Salmonella paratyphi-B*	14	11	14	14	10	14	20	19	12	18	18	-	18
*Salmonella typhimurium*	14	11	12	12	12	-	22	22	-	20	15	-	24
*Proteus vulgaris*	17	12	10	-	13	-	26	23	-	20	16	-	30
*Micrococcus luteus*	28	13	16	19	15	13	17	14	16	16	-	-	26
*Klebsiella pneumoniae*	20	10	12	-	10	18	24	20	12	22	14	-	20
*Shigella flexneri*	22	-	13	13	15	19	25	18	14	22	17	-	30
**Fungi**													C
*Candida albicans*	14	-	-	13	15	-	13	-	12	13	-	-	28
*Malassezia pachydermatis*	12	-	-	10	14	12	-	-	10	11	-	12	26

C-Streptomycin—standard antibacterial agent; C-Ketoconazole—standard antifungal agent.

**Table 4:** Minimum inhibitory concentration of the synthesized compounds (µg/mL).

Organism	5a	5c	5d	5f	5g	5h	5i	5j	5k	C
**Bacteria**										
*Enterobacter aerogenes*	250	250	500	-	62.5	125	250	-	62.5	25
*Staphylococcus aureus*	500	125	250	-	125	250	125	250	125	6.25
*Staphylococcus epidermidis*	250	250	250	125	31.25	125	500	250	-	25
*Staphylococcus aureus*-MRSA	250	125	125	250	31.25	62.5	250	250	125	6.25
*Salmonella paratyphi-B*	250	250	250	250	62.5	62.5	500	125	125	30
*Salmonella typhimurium*	250	500	500	-	62.5	62.5	-	62.5	250	6.25
*Proteus vulgaris*	125	500	-	-	31.25	62.5	-	62.5	125	6.25
*Micrococcus luteus*	31.25	125	62.5	250	125	250	125	125	-	6.25
*Klebsiella pneumoniae*	62.5	500	-	125	31.25	62.5	500	62.5	250	25
*Shigella flexneri*	62.5	250	250	62.5	31.25	125	250	62.5	125	6.25

**Table 5:** IC_50_ (µM) values of synthesized compounds against IMR90, A549, and HepG-2 cells.

Compound	A549	HepG-2	IMR90
5a	190 ± 1.35	275 ± 1.52	>250
5e	130 ± 0.65	164 ± 2.01	>250
5g	**72** ± 3.21	>300	>250
5h	**58** ± 2.31	**73** ± 3.88	>250
5i	134 ± 1.05	197 ± 2.34	>250
5j	111 ± 1.82	>300	>250
Cisplatin (µM)	16.4 ± 3.19	22.1 ± 3.08	NT

NT—not tested.

**Table 6:** Binding energy of synthesized compounds.

Compound	Binding Energy (kcal/mol) ^a^	
	DNA Topoisomerase IV (4EMV)	Anaplastic Lymphoma Kinase (2XP2)
**5a**	−7.31	−6.55
**5b**	−8.64	NC
**5c**	−8.76	NC
**5d**	−8.84	NC
**5e**	−7.63	−7.79
**5f**	−8.93	NC
**5g**	−8.06	−8.12
**5h**	−7.24	−7.10
**5i**	−8.98	−8.28
**5j**	−7.63	−8.72
**5k**	−8.75	NC
**5l**	−7.71	NC
CL	−9.80	−8.42

^a^ Calculated using AutoDock; NC: Not calculated, CL: Co-crystallized ligand with receptor.

## Supplementary Materials

The supplementary materials are available at https://www.mdpi.com/1420-3049/24/19/3501/s1.
