# Supplementary material for: A New Class of β–Pyrrolidino-1,2,3-Triazole Derivatives as β-Adrenergic Receptor Inhibitors: Synthesis, Pharmacological, and Docking Studies
Source: Molecules. 2019 Sep 26;24(19):3501. doi: 10.3390/molecules24193501 (PMC6803965; doi:10.3390/molecules24193501)
Supplement: Supplementary file 1 [file molecules-24-03501-s001.pdf]

Supporting Information

Investigation on a new class of β–pyrrolidino-1,2,3-triazole derivatives as β-adrenergic receptor inhibitors: Synthesis, pharmacological and docking studies

Kaliyappan Easwaramoorthi[1,2], Jeya A. Rajendran[1*], Kella Chennakesava Rao[2,4], Chandrasekar Balachandran[3], Yuvaraj Arun[4], Sakkarapalayam M. Mahalingam[5], Natarajan Arumugam[6], Abdulrahman I. Almansour[6], Raju Suresh Kumar[6], Dhaifallah M. Al-thamili[6], Shin Aoki[7]

[1]Department of Chemistry, Loyola College, Chennai-600034, TN, India.
[2]R&D Centre, Malladi Drugs & Pharmaceuticals Ltd, Chennai-600124, TN, India.
[3] Faculty of Pharmaceutical Sciences, Tokyo University of Science, 2641 Yamazaki, Noda 278-8510 Japan.
[4]Organic & Bioorganic Chemistry Laboratory, CSIR-Central Leather Research Institute, Adyar, Chennai-600020, TN, India.
[5] Department of Chemistry, SRM institute of Science and Technology, Kattankulathur, Kancheepuram 603203, India.
[6]Department of Chemistry, College of Science, King Saud University, P.O. Box 2455, Riyadh 11451, Saudi Arabia.
[7]Research Institute of Science and Technology, Tokyo University of Science, 2641 Yamazaki, Noda 278-8510 Japan.

General

Chemistry

Melting points were determined using a Mettler Toledo melting point apparatus by open capillary tube method. Thin layer chromatography (TLC) was conducted using pre-coated Merck TLC Silicagel 60 F254 and spots were detected using UV light at 254 nm. IR spectra were recorded by KBr pellet method on Shimadzu Prestige 21 FTIR instrument in the range of 4000 to 400 cm-1. 1H NMR and 13C NMR spectra were recorded using Ultrashield-400 & 100 MHz, Bruker, SWZ spectrometer using DMSO-*d6* as a solvent and TMS as an internal standard. Chemical shifts (δ) were reported, in ppm (parts per million). The following abbreviations are used: s-singlet, d-doublet, t-triplet and m-multiplet. Low resolution mass spectra were measured on Agilent 6110 LCMS 2

mass spectrometer using ESI mode. The elemental analysis was done on Thermo Fischer Flash 1112 series elemental analyzer. The purities of the synthesized novel β-pyrrolidino-1,2,3-triazoles are obtained using LC-2010AHT, LC-10ATVP with SPD-M10 (DAD detector) with Inertsil ODS-3V-250 x 4.6 mm-5.0 μm HPLC column and sodium dihydrogen phosphate buffer with acetonitrile as mobile phase and CLASS-VP as chromatography data processor. The chiral purity of the novel β-pyrrolidino-1,2,3-triazoles have been obtained with Chiralpak-ADH-250 x 4.6 mm, 5.0 μm HPLC chiral column using 1% ethanol in *n*-hexane with 0.1% of diethylamine at a flow rate of 1.0 mL/min using UV variable wavelength detector.

HPLC method:

The purities of the synthesized novel β-pyrrolidino-1,2,3-triazoles are obtained using LC-2010AHT, LC-10ATVP with SPD-M10 (DAD detector) with Inertsil ODS-3V-250 x 4.6 mm-5.0μm HPLC column and sodium dihydrogen phosphate buffer with acetonitrile as mobilephase and CLASS-VP as chromatography data processor. The chiral purity of the novel β-pyrrolidino-1,2,3-triazoles have been obtained with Chiralpak-ADH-250 x 4.6 mm, 5.0 μm HPLC chiral column using 1% ethanol in *n*-hexane with 0.1% of diethylamine at a flow rate of 1.0 mL/min using UV variable wavelength detector.

Microbial organisms

The following bacteria and fungi were used for the experiment. Bacteria: Salmonella paratyphi-B, Klebsiellapneumoniae MTCC 109, Shigellaflexneri MTCC 1457, Staphylococcus epidermidis MTCC 3615, Enterobacter aerogenes MTCC 111, Salmonella typhimurium MTCC 1251, Micrococcus luteus MTCC 106, Proteus vulgaris MTCC 1771, Staphylococcus aureus (MRSA- methicillin resistant) and Staphylococcus aureus MTCC 96. The reference cultures were obtained from Institute of Microbial Technology (IMTECH), Chandigarh, India-160 036; fungi: Candida albicans MTCC 227 and Malassesiapachydermatis. All the other tested cultures were obtained from the Department of Microbiology, Christian Medical College, Vellore, Tamil Nadu, India.

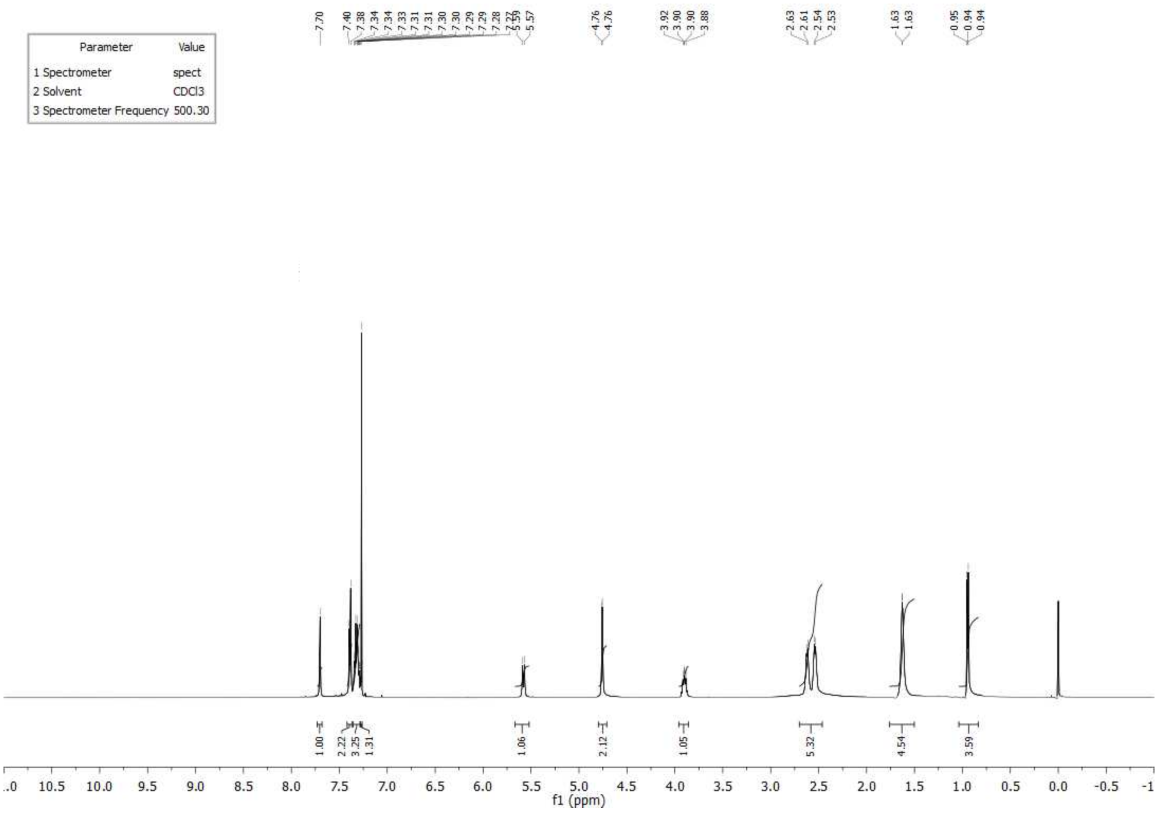

Figure 1. ^{1}H NMR spectrum of **5a**

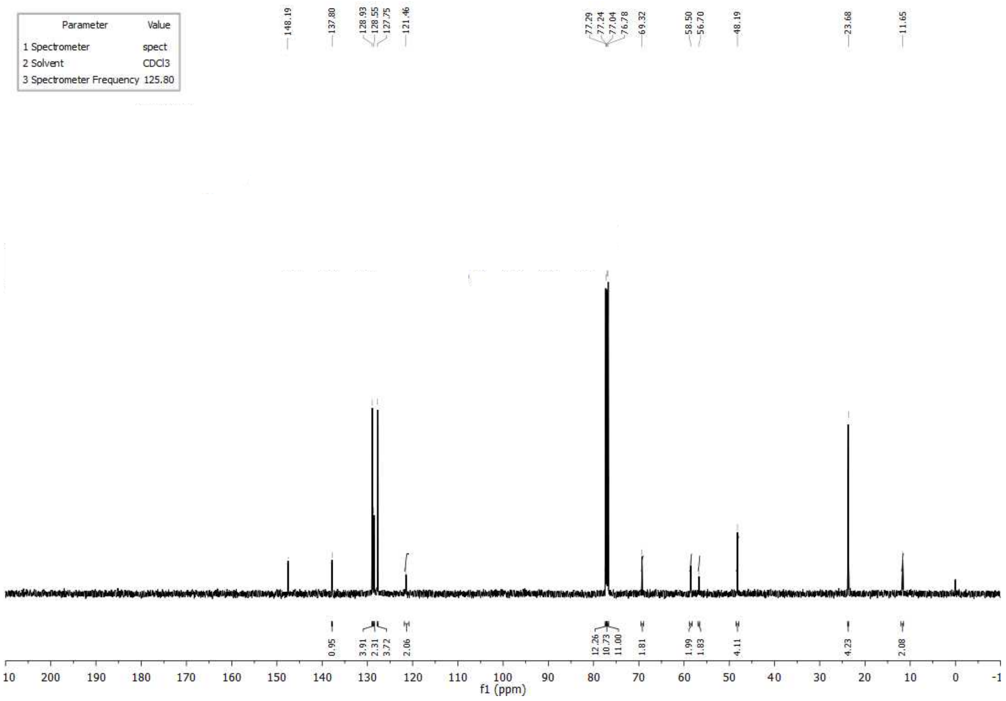

Figure 2. ^{13}C NMR spectrum of **5a**

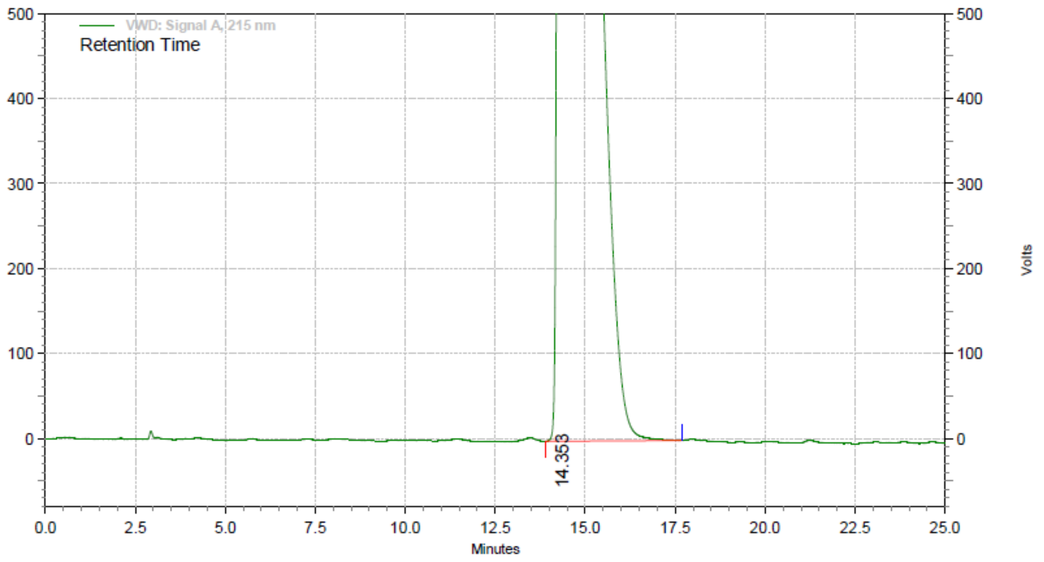

Figure 3. Chiral HPLC chromatogram of compound **5a**

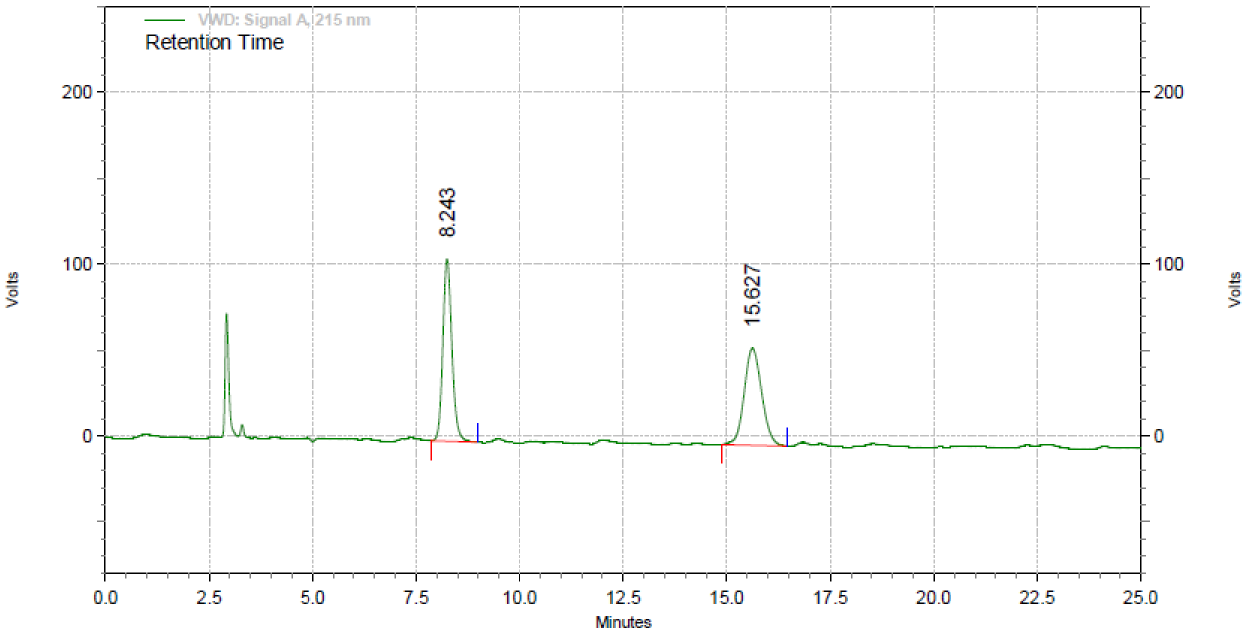

Figure 4. Chiral HPLC chromatogram of racemic compound **5a**

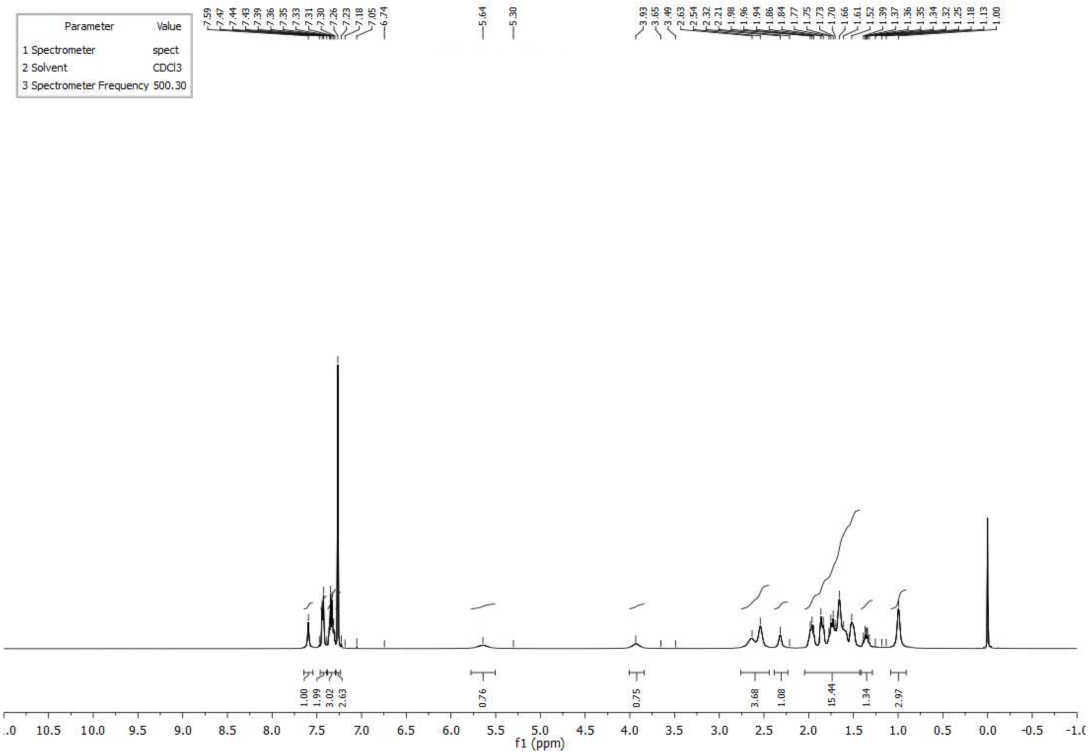

Figure 5. ^{1}H NMR spectrum of **5b**

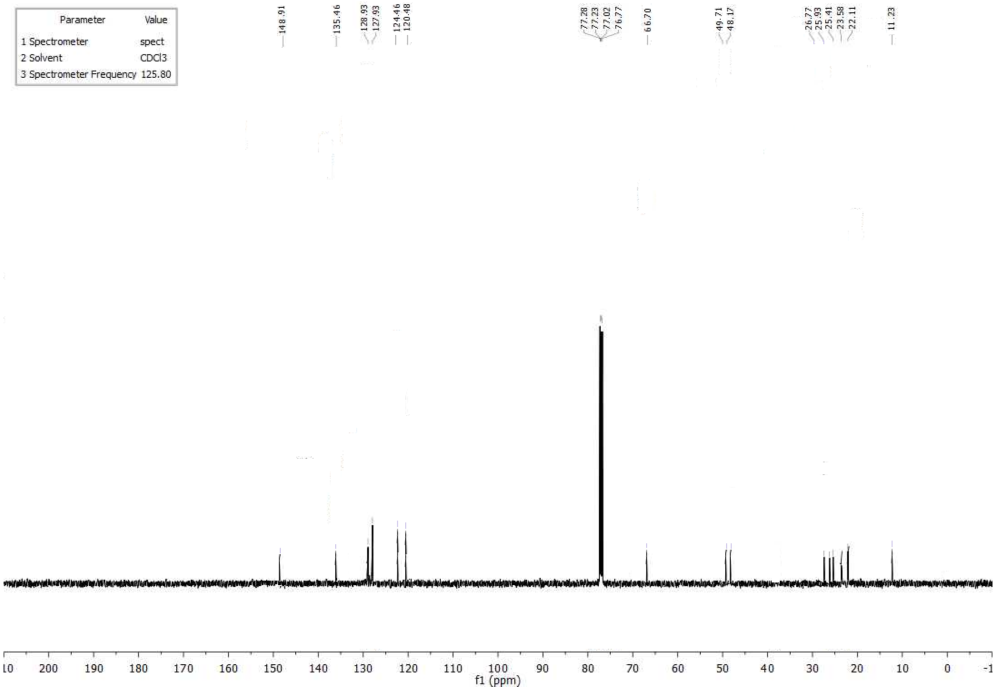

Figure 6. ^{13}C NMR spectrum of **5b**

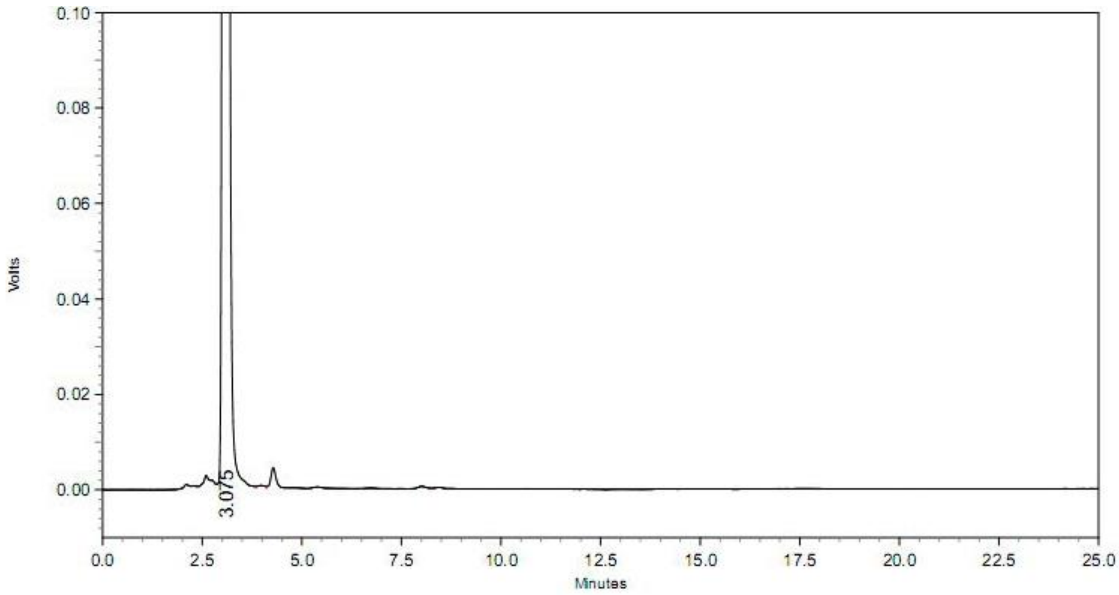

Figure 7. Chiral HPLC chromatogram of compound **5b**

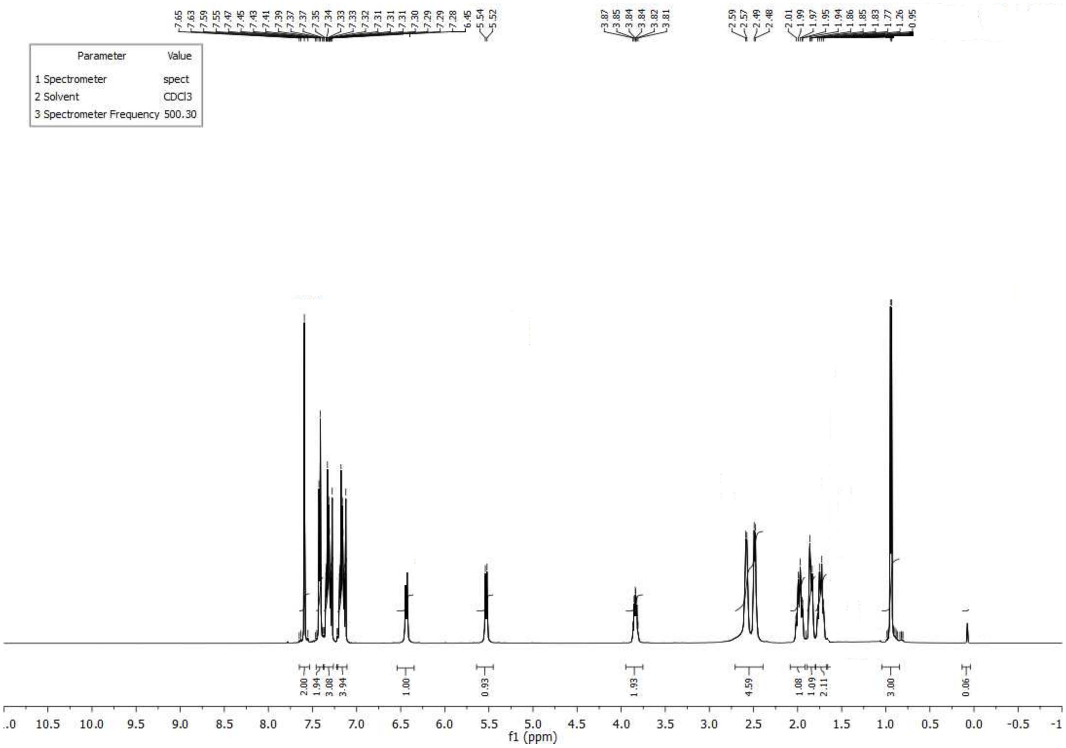

Figure 8. ^{1}H NMR spectrum of **5c**

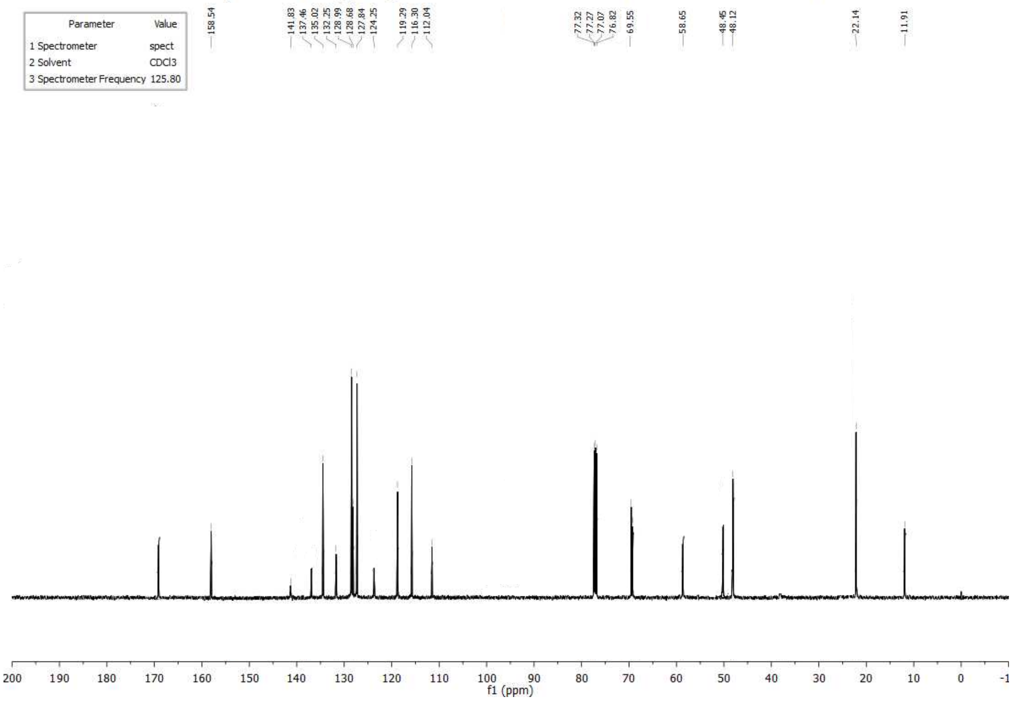

Figure 9. ^{13}C NMR spectrum of **5c**

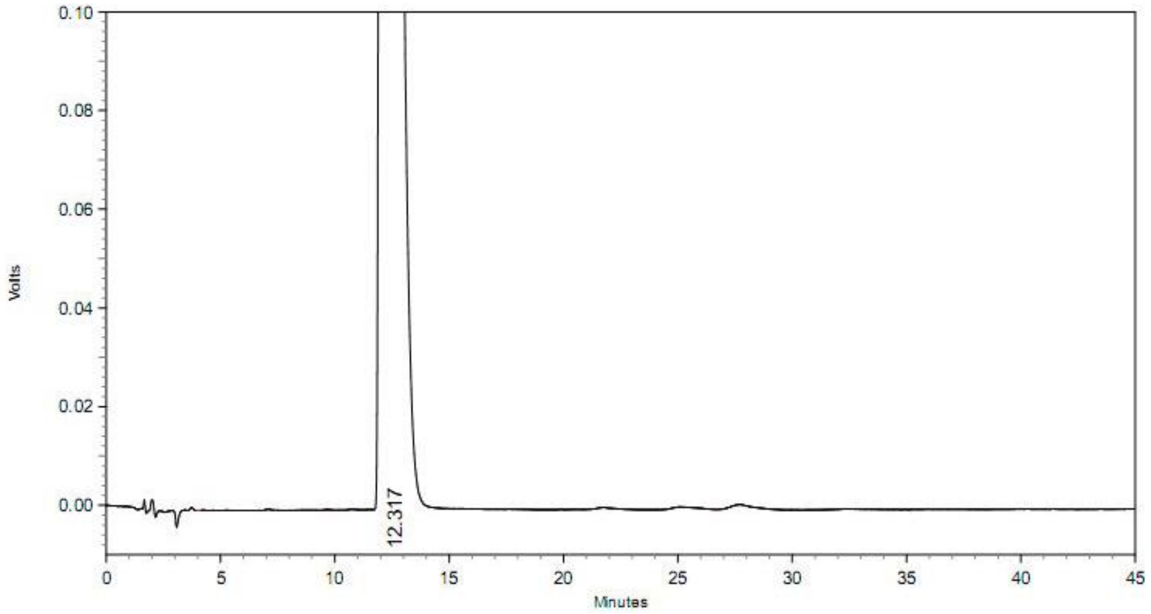

Figure 10. Chiral HPLC chromatogram of compound **5c**

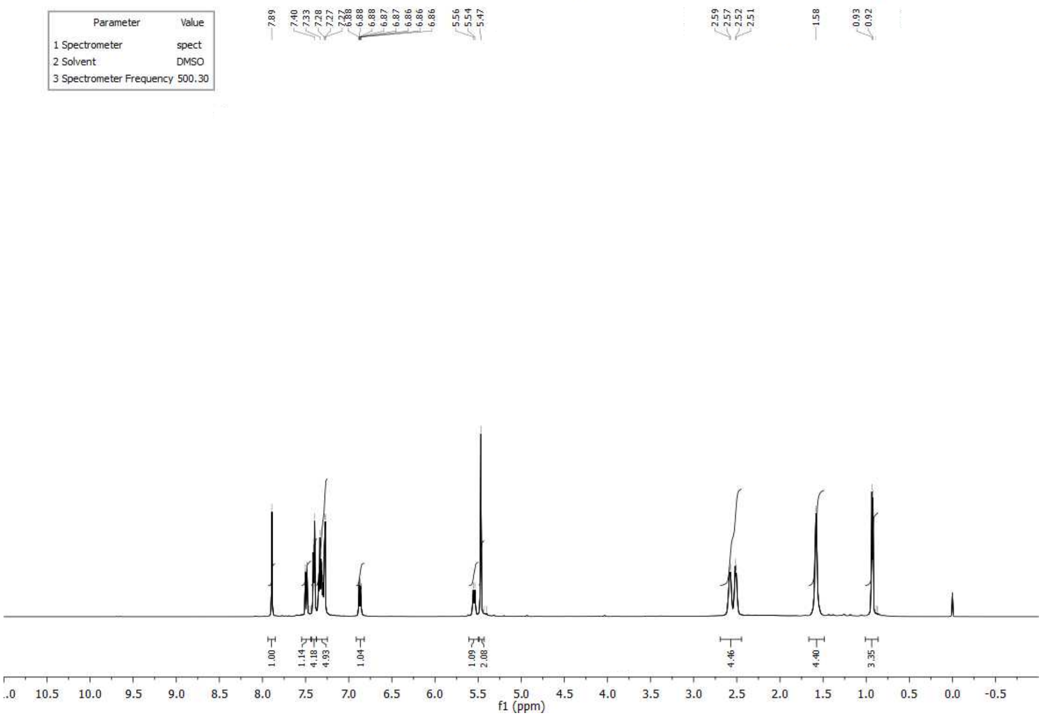

Figure 11. ^{1}H NMR spectrum of **5d**

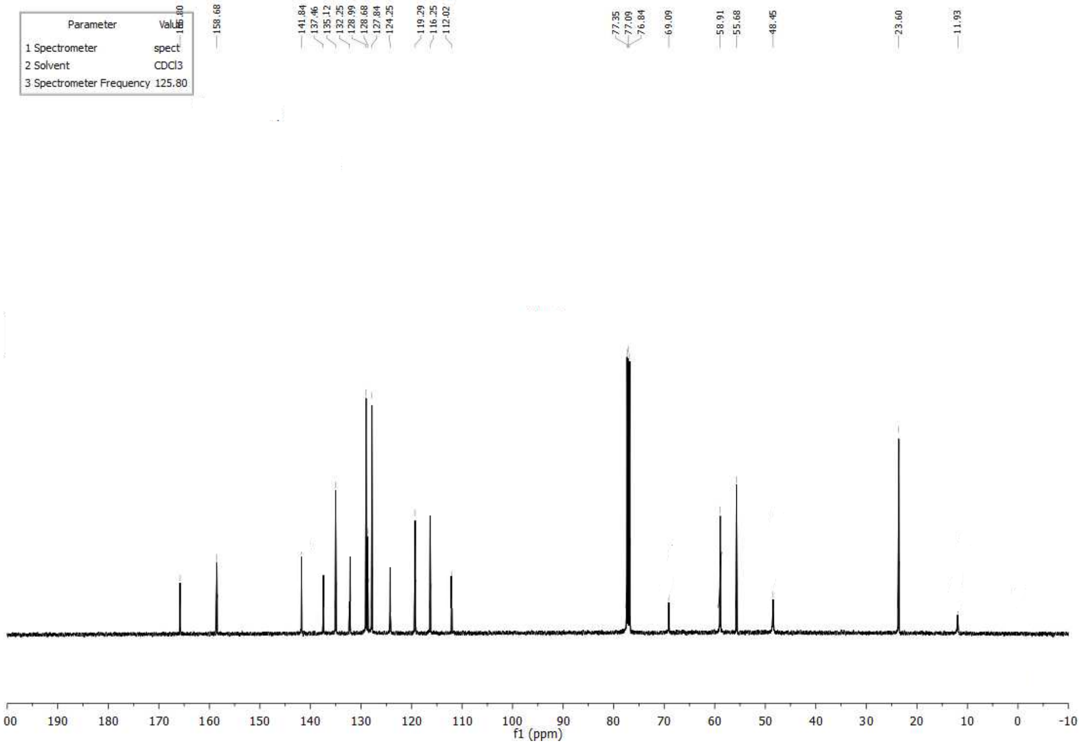

Figure 12. ^{13}C NMR spectrum of **5d**

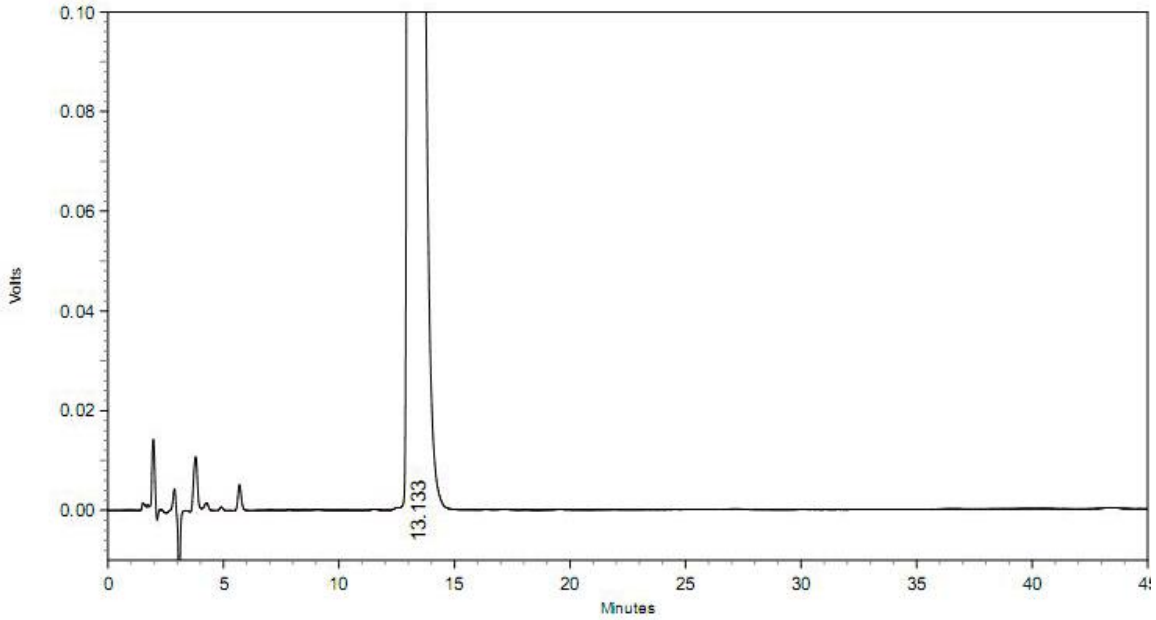

Figure 13. Chiral HPLC chromatogram of compound **5d**

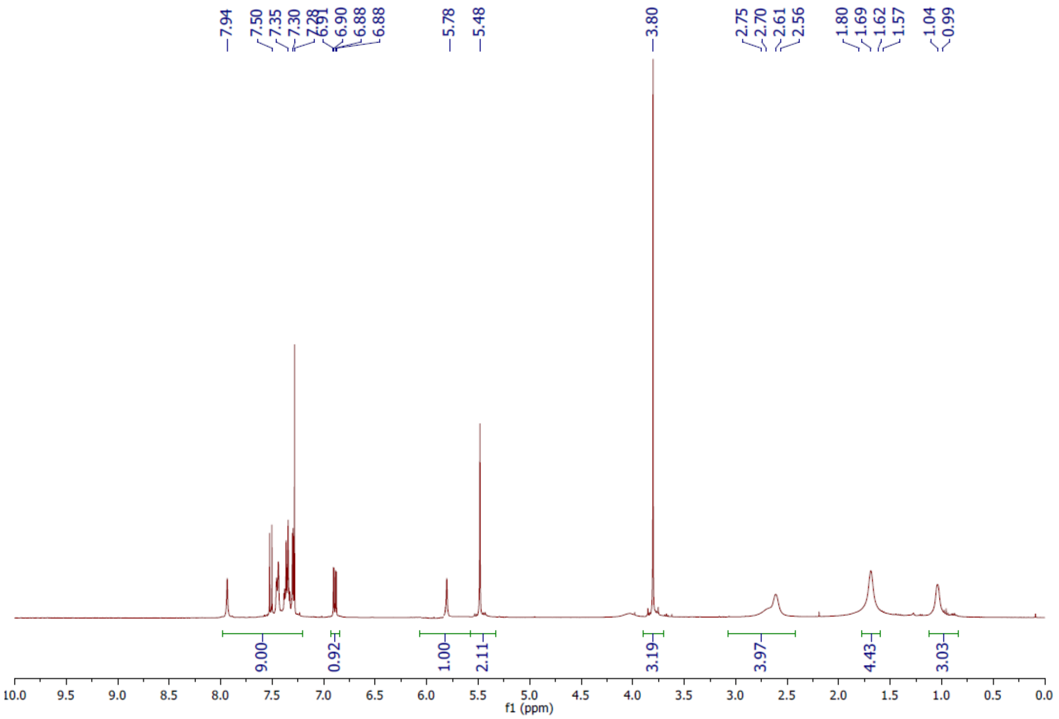

Figure 14. ^{1}H NMR spectrum of **5e**

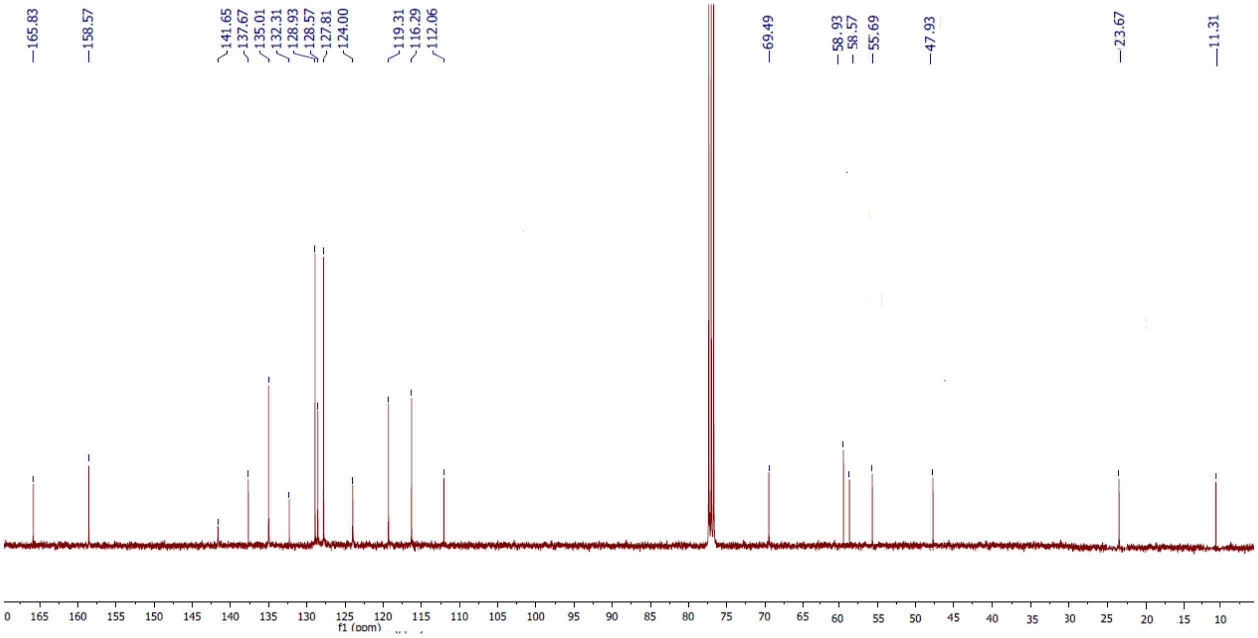

Figure 15. ^{13}C NMR spectrum of **5e**

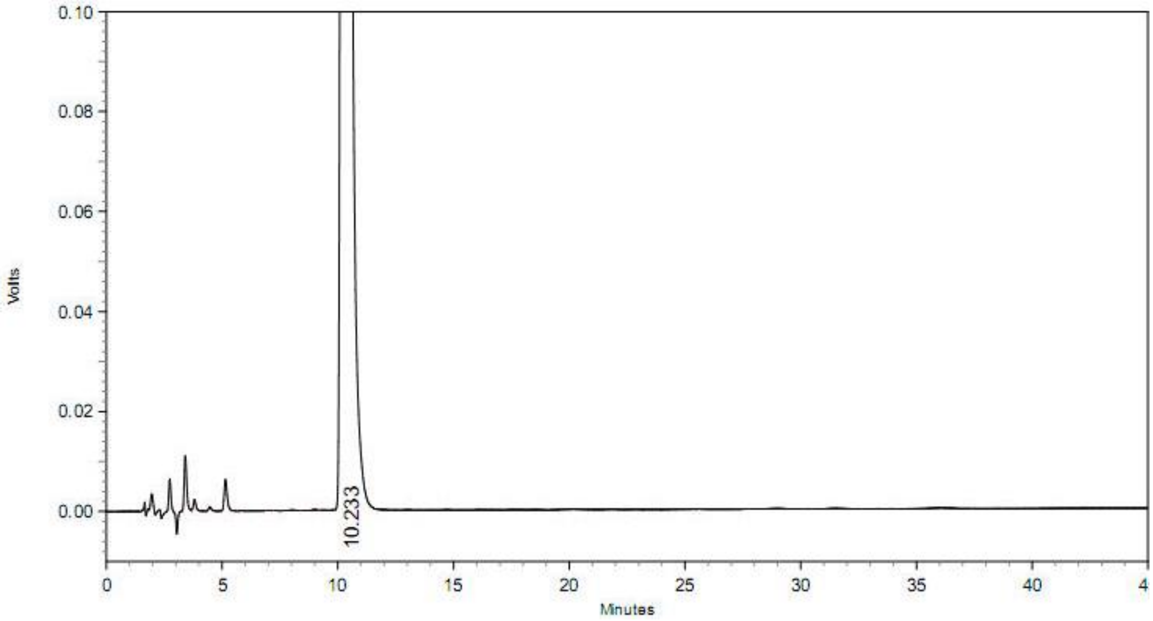

Figure 16. Chiral HPLC chromatogram of compound **5e**

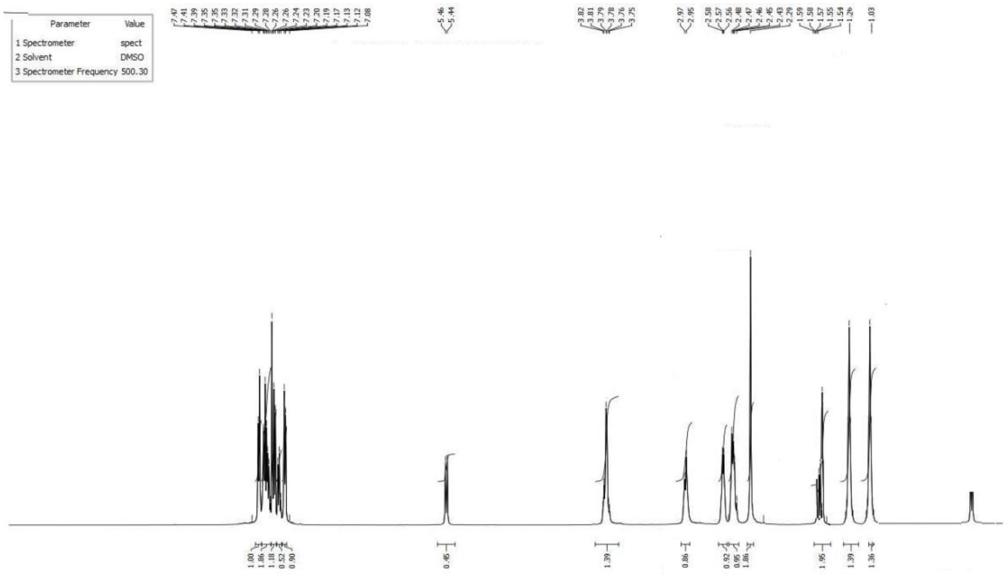

Figure 17. ^{1}H NMR spectrum of **5f**

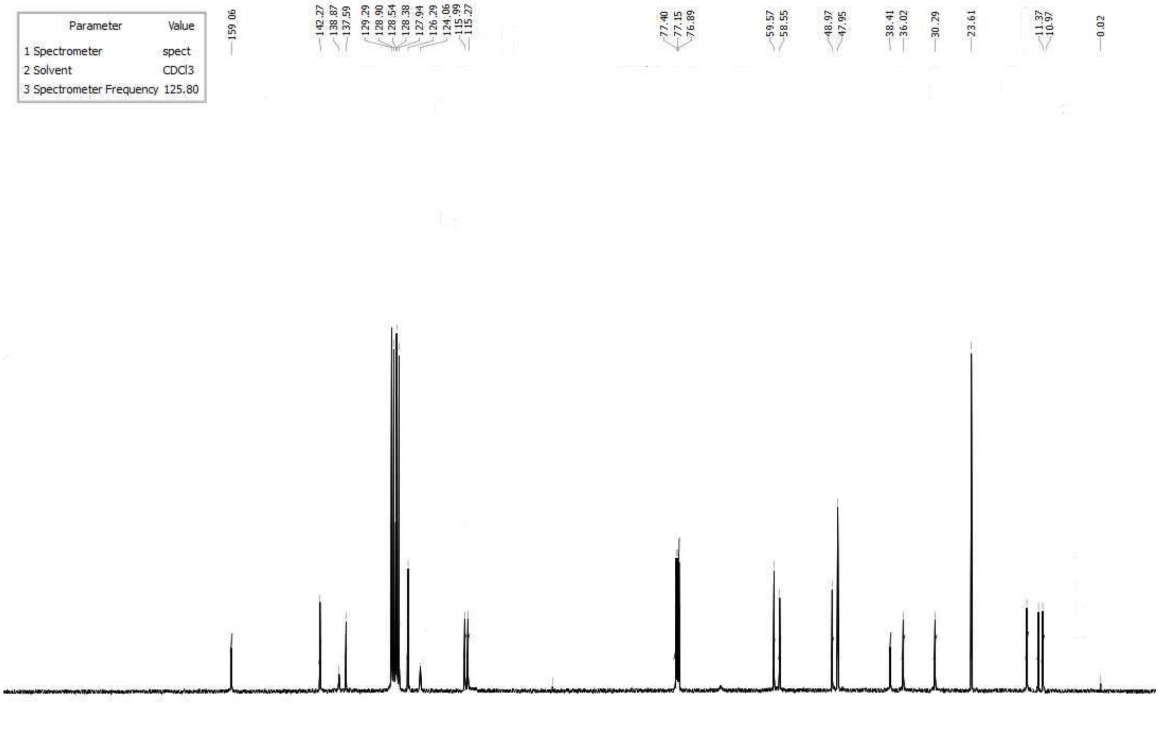

Figure 18. ^{1}H NMR spectrum of **5f**

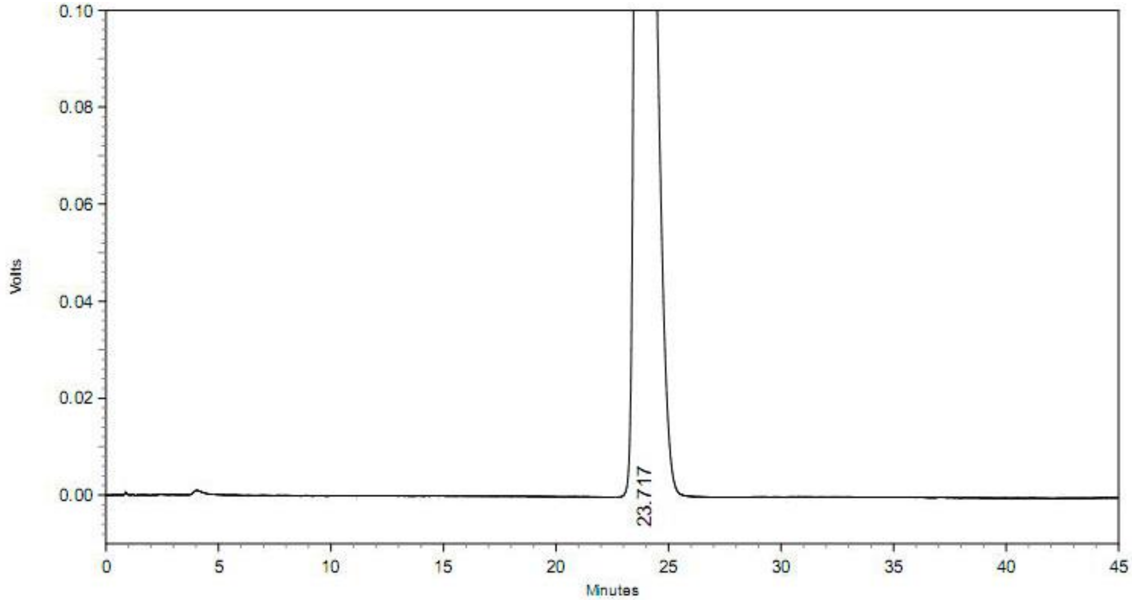

Figure 19. Chiral HPLC chromatogram of compound **5f**

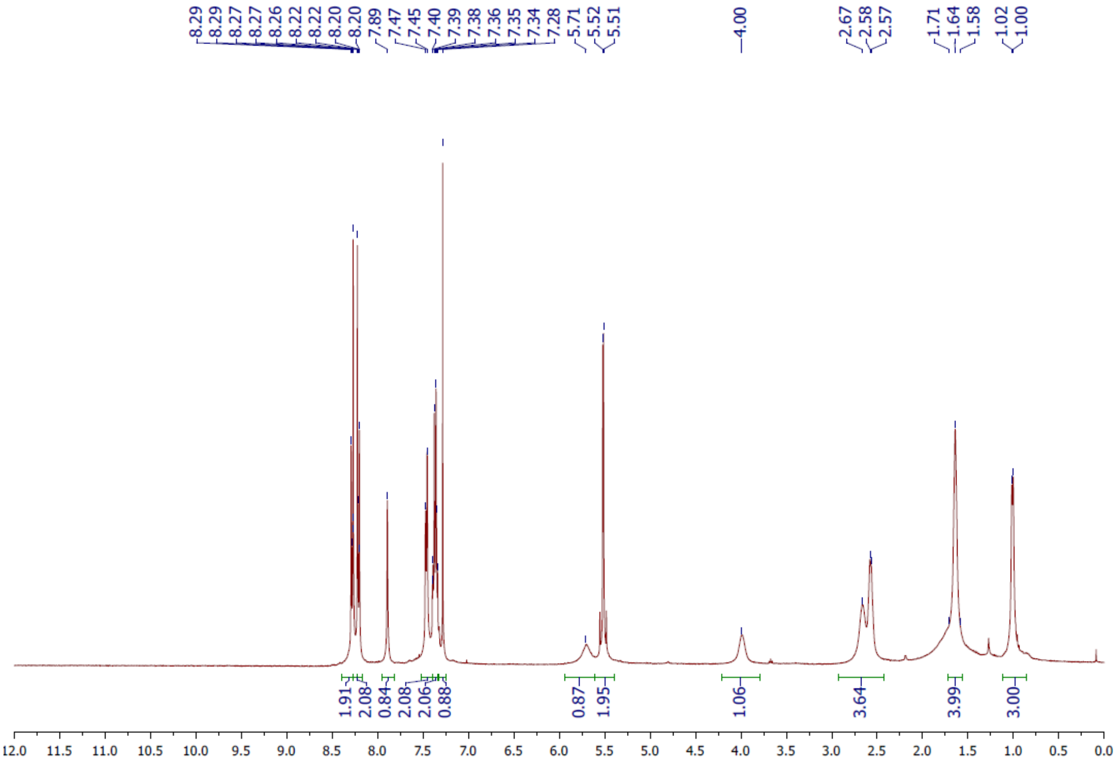

Figure 20. ^{1}H NMR spectrum of **5g**

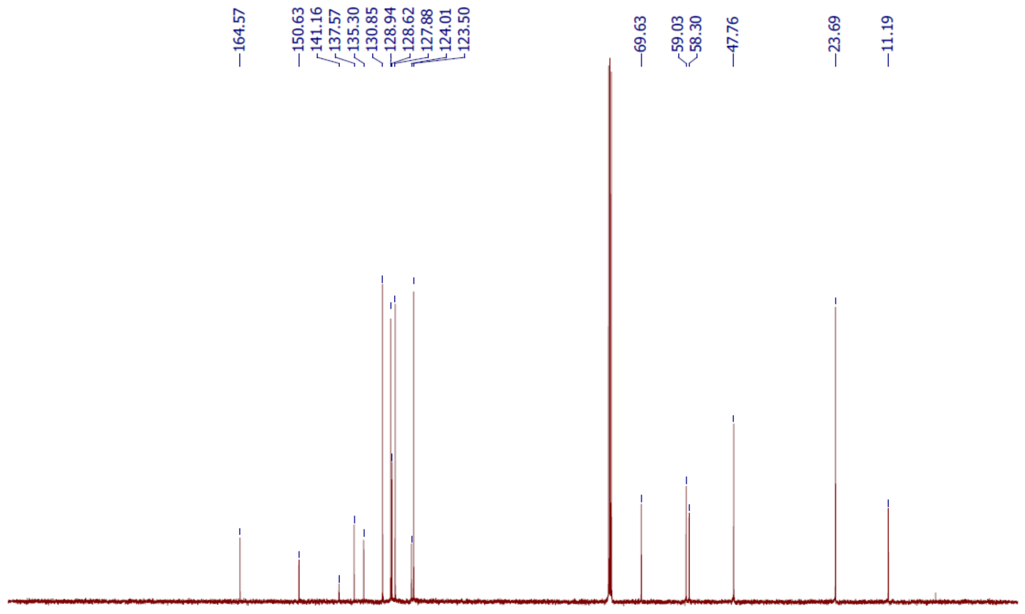

Figure 21. ^{13}C NMR spectrum of **5g**

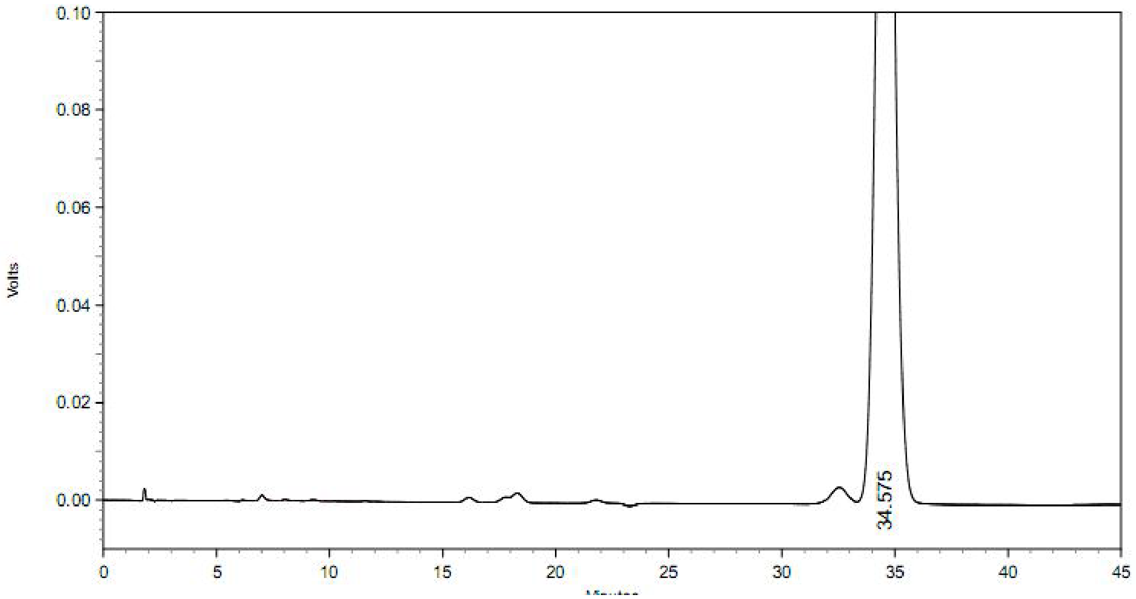

Figure 22. Chiral HPLC chromatogram of compound **5g**

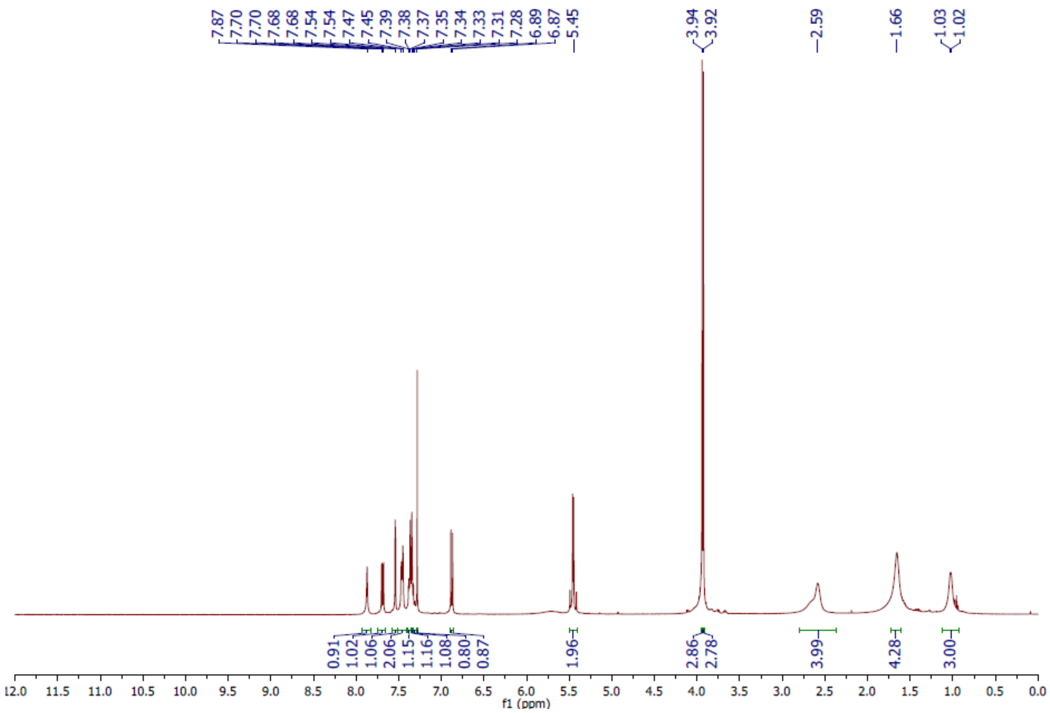

Figure 23. ^{1}H NMR spectrum of **5h**

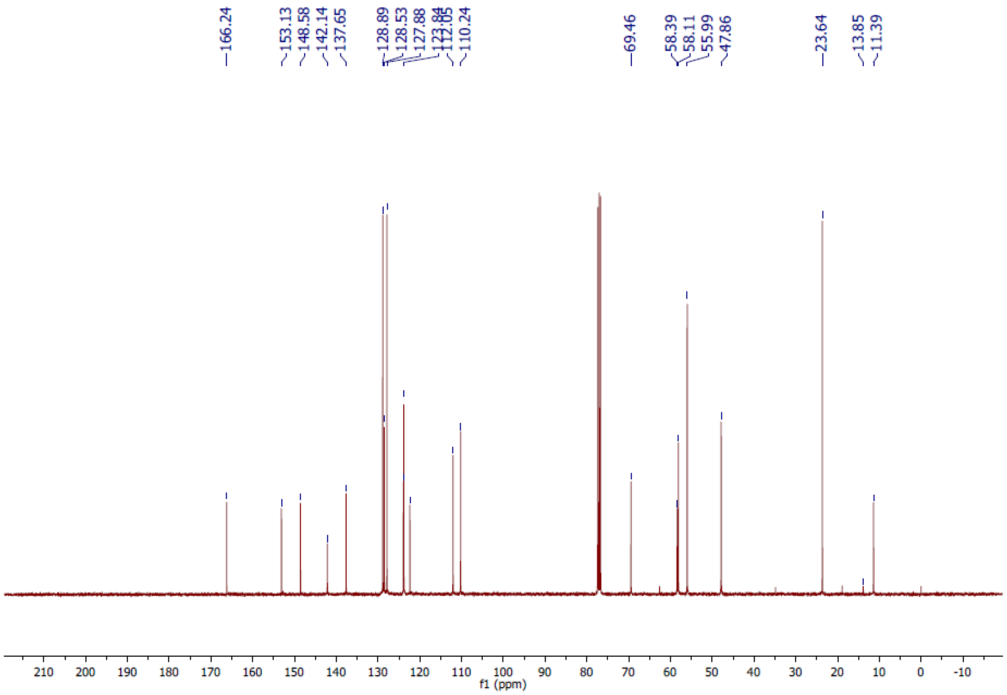

Figure 24. ^{13}C NMR spectrum of **5h**

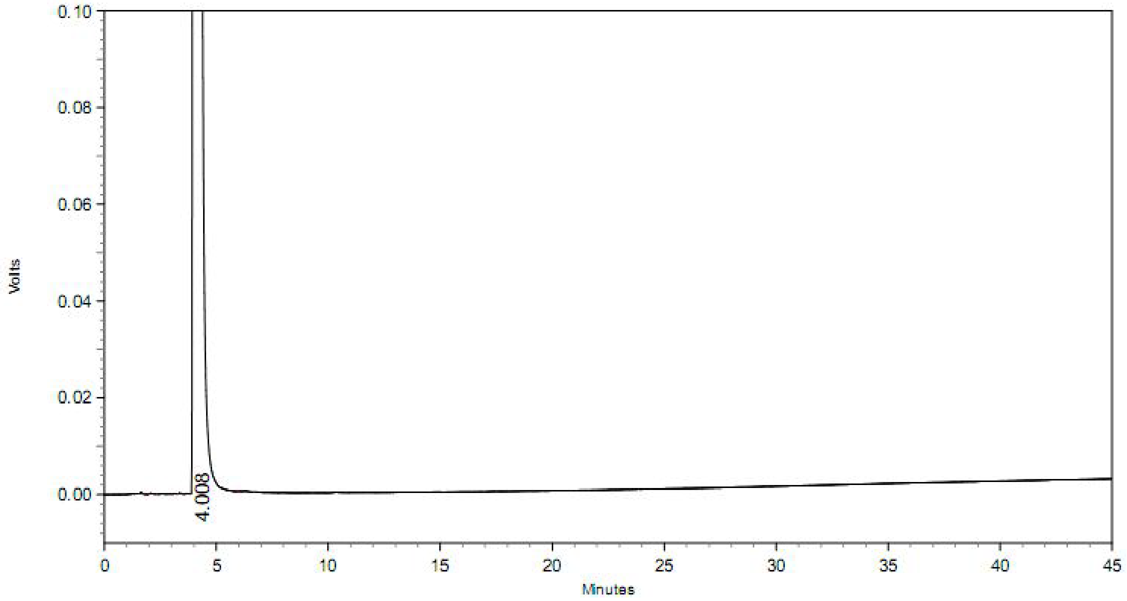

Figure 25. Chiral HPLC chromatogram of compound **5g**

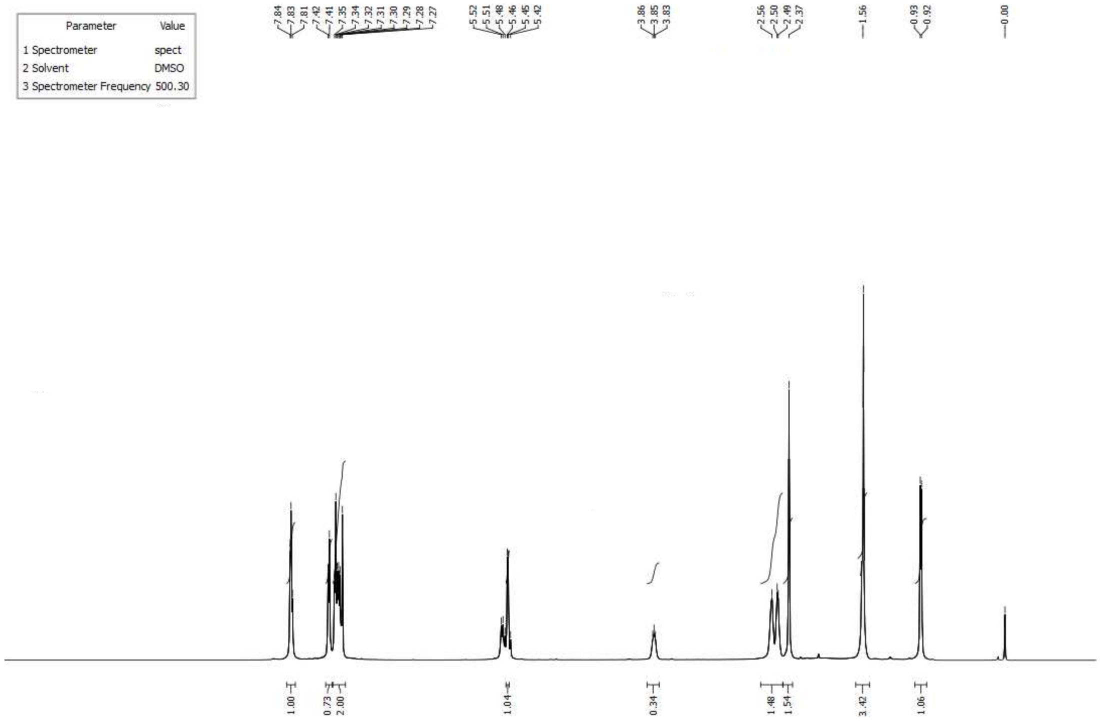

Figure 26. ^{1}H NMR spectrum of **5i**

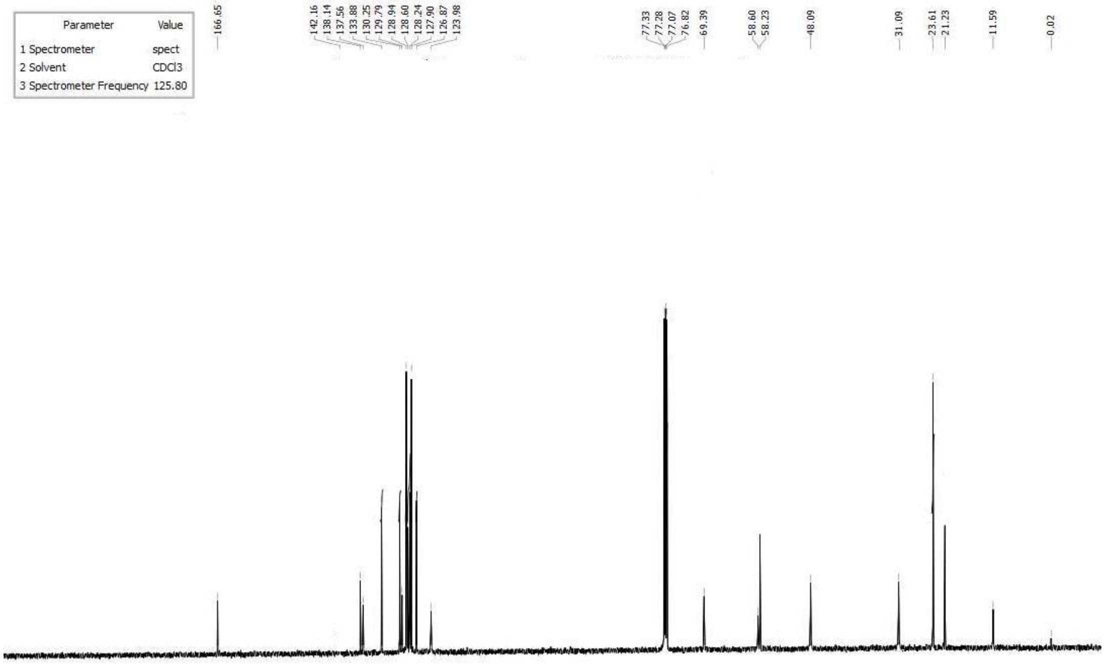

Figure 27. ^{13}C NMR spectrum of **5i**

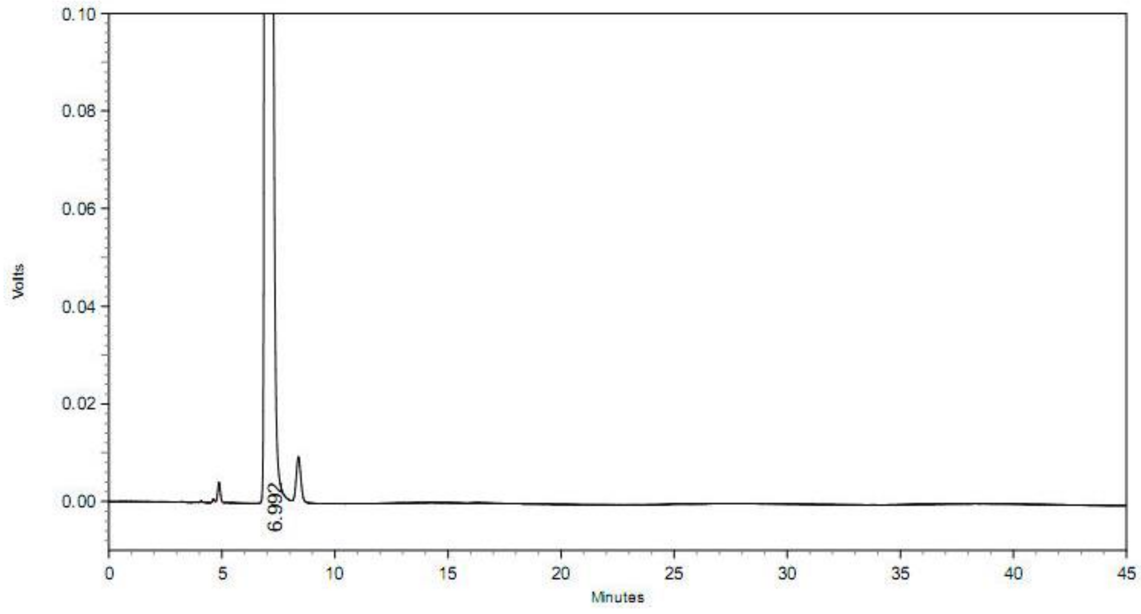

Figure 28. Chiral HPLC chromatogram of compound **5i**

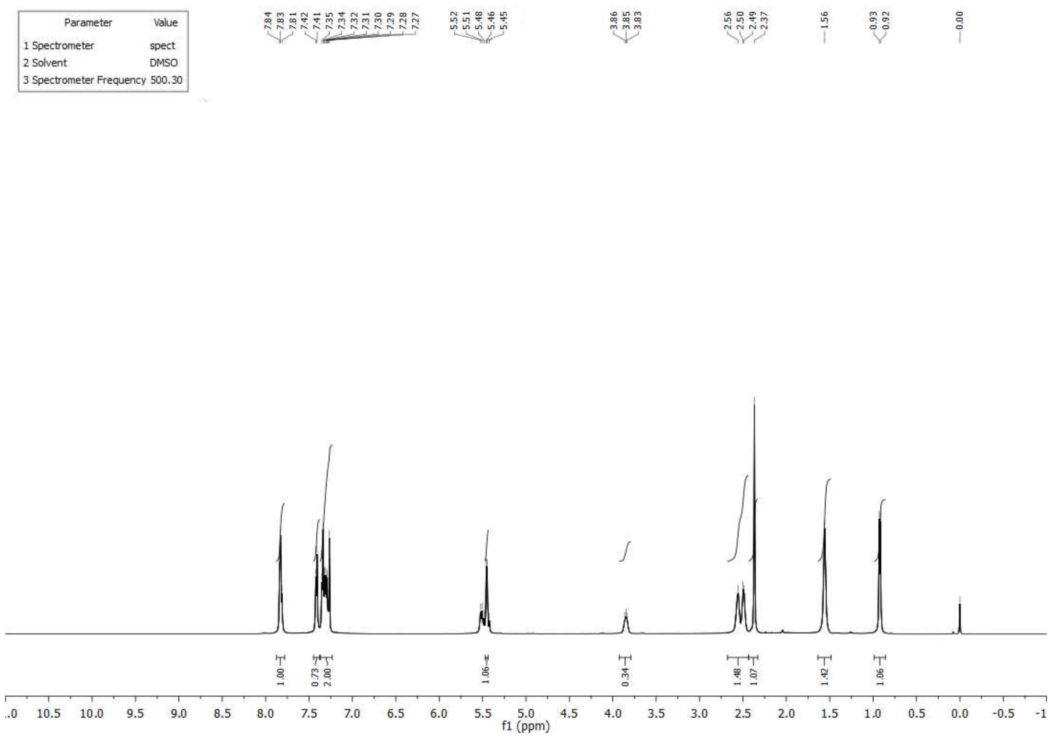

Figure 29. ^{1}H NMR spectrum of **5j**

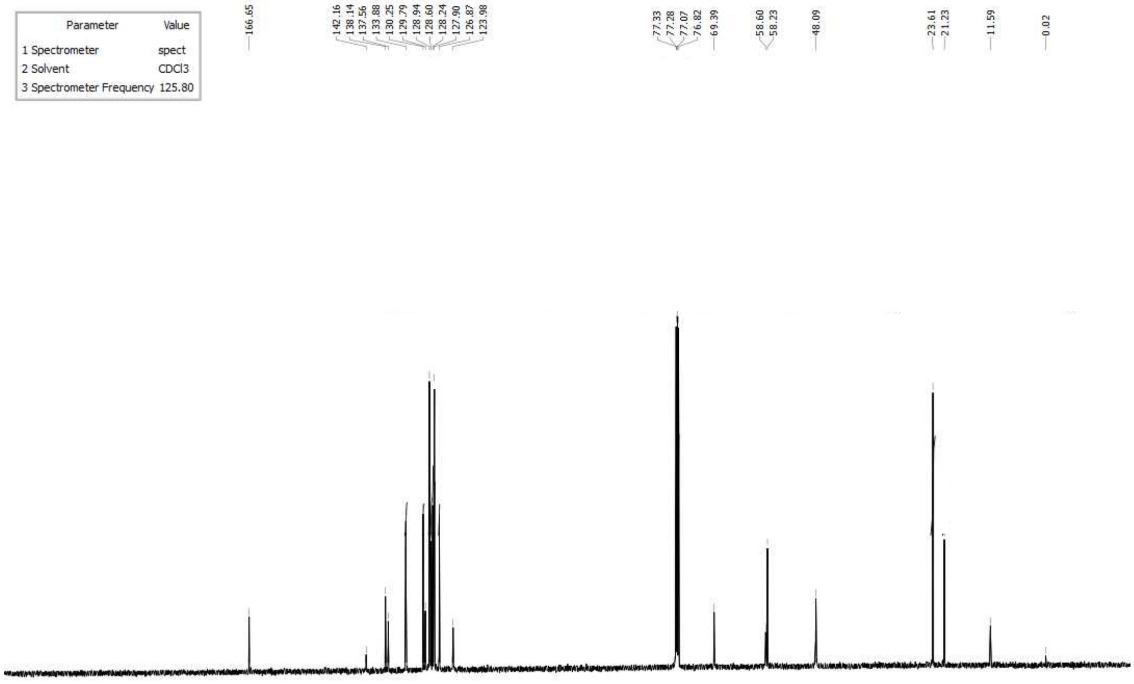

Figure 30. ^{13}C NMR spectrum of **5j**

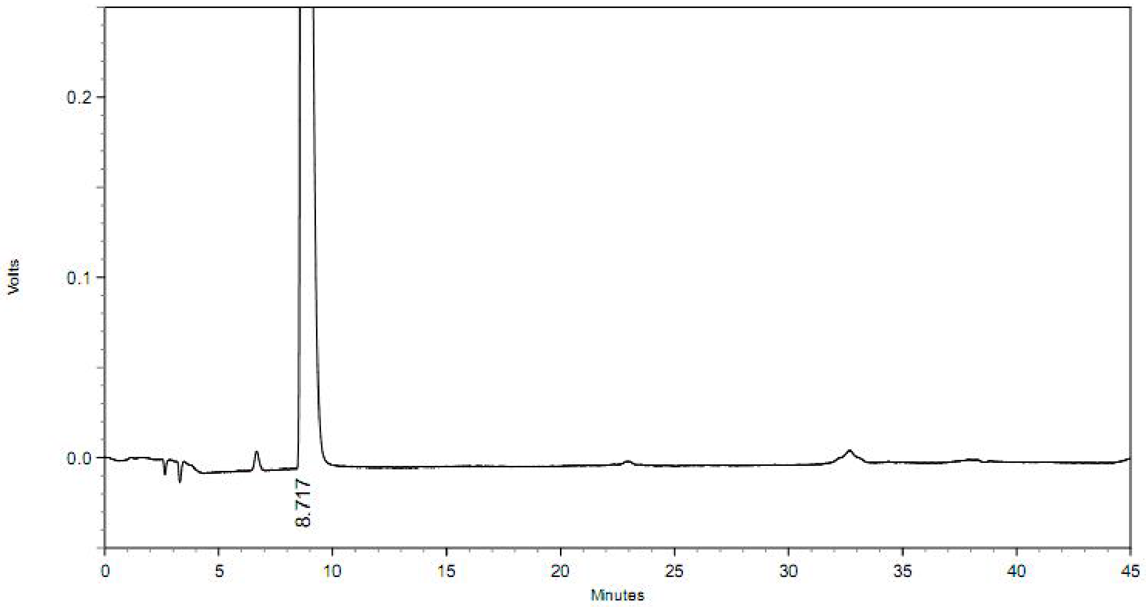

Figure 31. Chiral HPLC chromatogram of compound **5j**

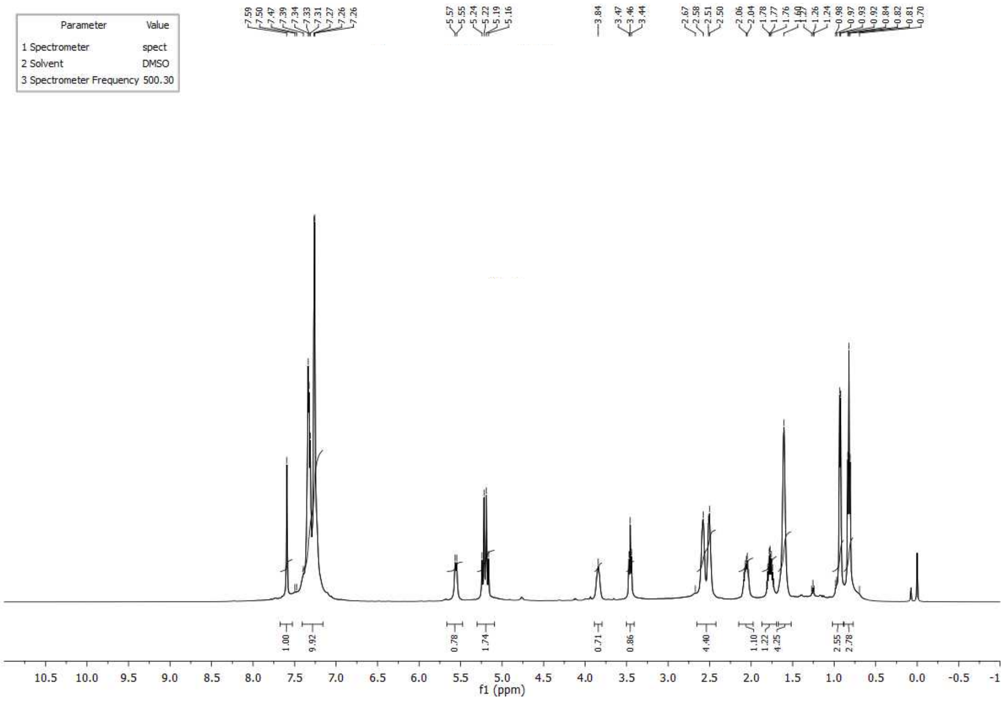

Figure 32. ^{1}H NMR spectrum of **5l**

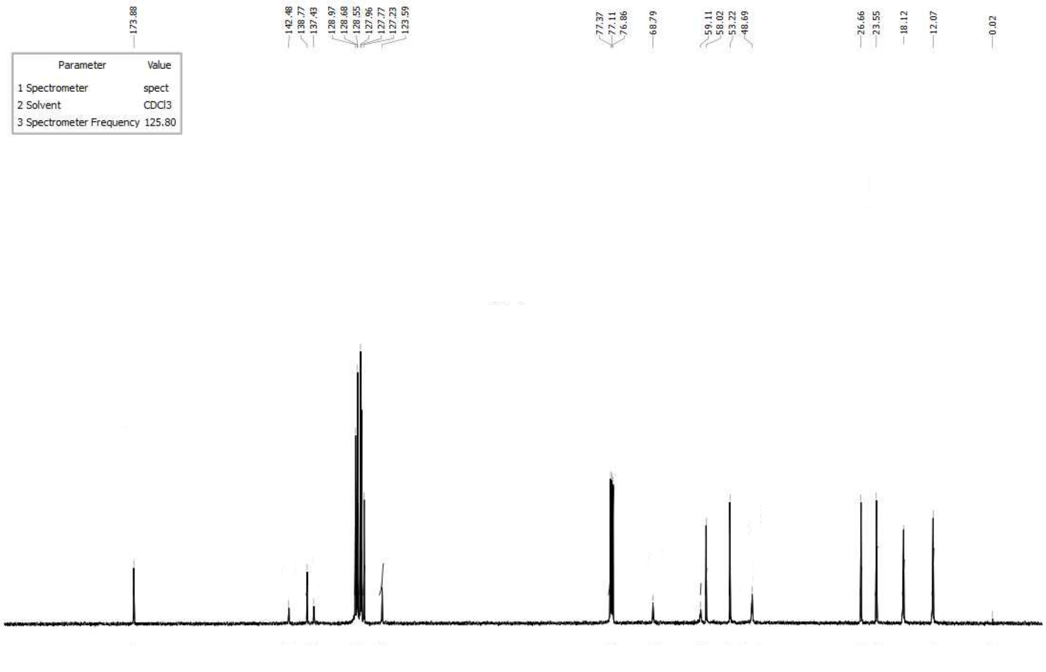

Figure 33. ^{13}C NMR spectrum of **5l**

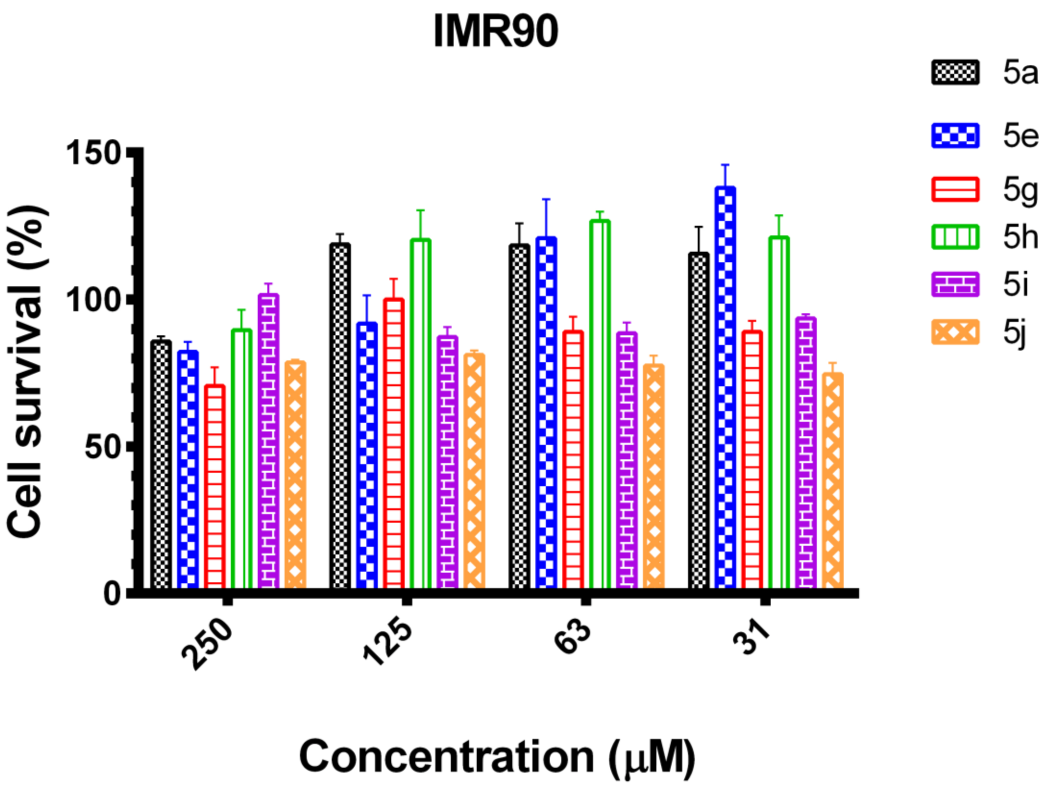

Figure 34. Cytotoxic studies of triazole derivatives with normal cell lines

Molecular docking studies *(Experimental)*

In silico docking studies were executed using the AutoDock Tools (ADT) version 1.5.6 and AutoDock version 4.2.5.1 docking package.[1] The DNA topoisomerase IV and Anaplastic Lymphoma Kinase structures were obtained from the Protein Data Bank (PDB ID: 4EMV and 2XP2 respectively).[2] The co-crystallized ligands were extracted from the receptors. Then, the polar hydrogen atoms were added to the receptor structures. Lower occupancy residue structures were deleted and any incomplete side chains were replaced using the ADT. Crystal waters were removed, Gasteiger charges were added to each atom, and merged the non-polar hydrogen atoms to the receptor structures. The

distance between donor and acceptor atoms for the hydrogen bond formation was defined as 1.9 Å with a tolerance of 0.5 Å, and the acceptor– hydrogen–donor angle was fixed to not less than 120˚. For further studies in ADT, the structures were exported to the PDBQT file format. The grid boxes with dimension of 40 × 40 × 40 Å^{3}and 60 × 60 × 60 Å^{3}with 0.375 Å spacing and centered on 14.860, 29.555, 6.941 and 29.697, 47.794, 8.863was generated around the binding site of co-crystallized ligand on 4EMV and 2XP2 respectively. The grid energy calculations were carried out by setting the co-crystallized ligand center as the center of the box. The bound ligands were extracted from the complexes and free energy of binding was estimated to verify the reproducibility of the docking experiments. This reproduced top scoring conformation falling within RMSD value of 0.58 to 1.53 Å and 1.63 to 1.95 Å with bound X-ray conformation for 4EMV and 2XP2 respectively, suggesting this technique is valid to study the docking of other compounds. After the validation study, the same protocol was applied for the synthesized compounds. Docking for each of the test molecules, were taken into 2.5 million energy evaluations. Twenty docked conformations were generated for each compound. Genetic algorithms was used to estimate the free energy of binding. Docked ligand and receptor conformations were analyzed in terms of energy, hydrogen bonding, polar and hydrophobic interactions. Complete studies of the ligand-receptor interactions were performed, and final coordinates of the ligand and receptor were saved. The free energy of binding (FEB) of all the synthesized compounds were estimated from the docking scores.

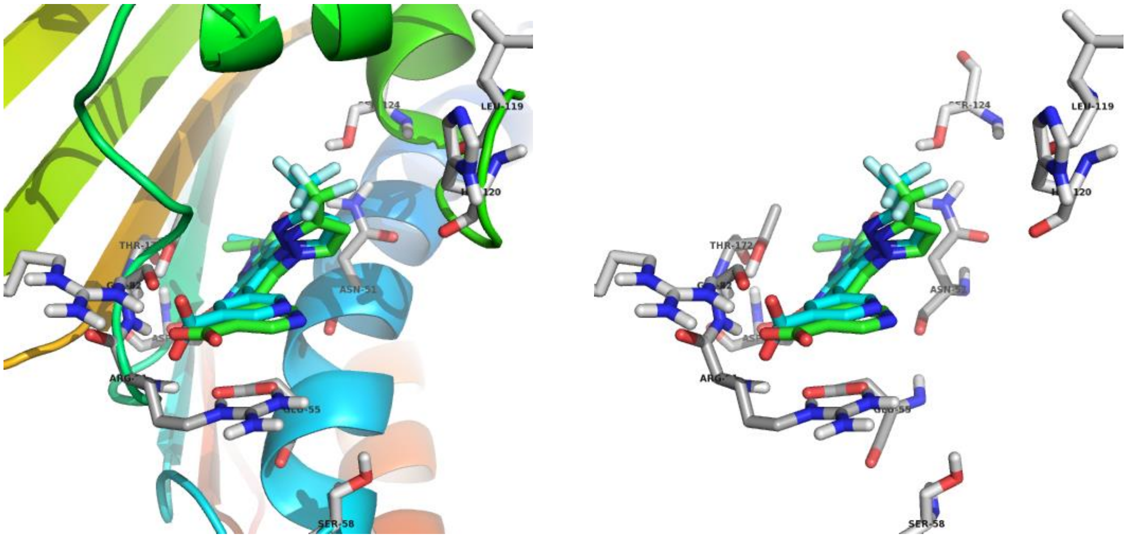

Method validation using crystallised and docked ligand with 4EMV receptor

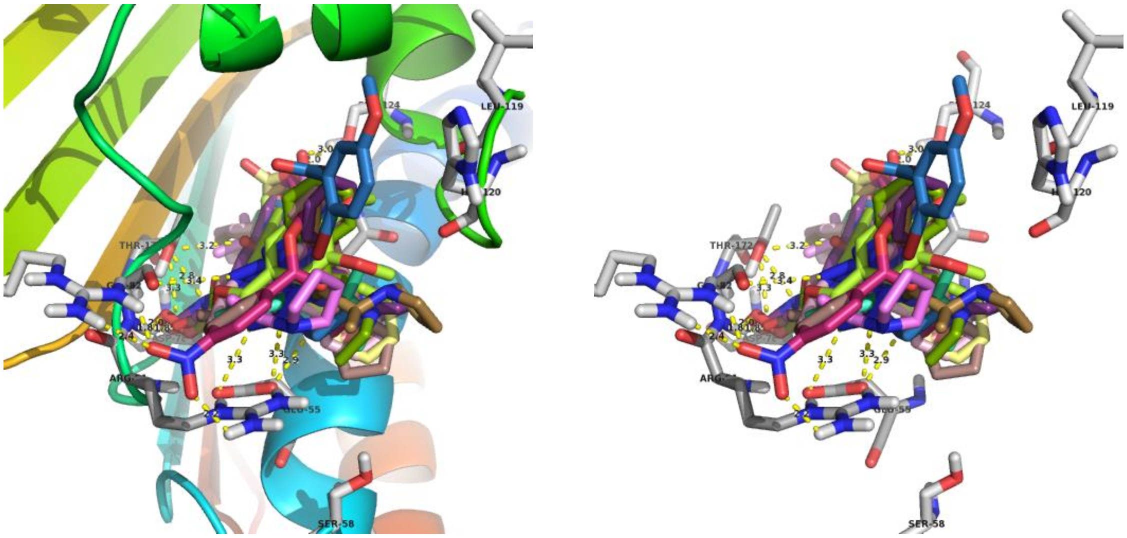

Docking mode of all the compounds in the active site of 4EMV receptor

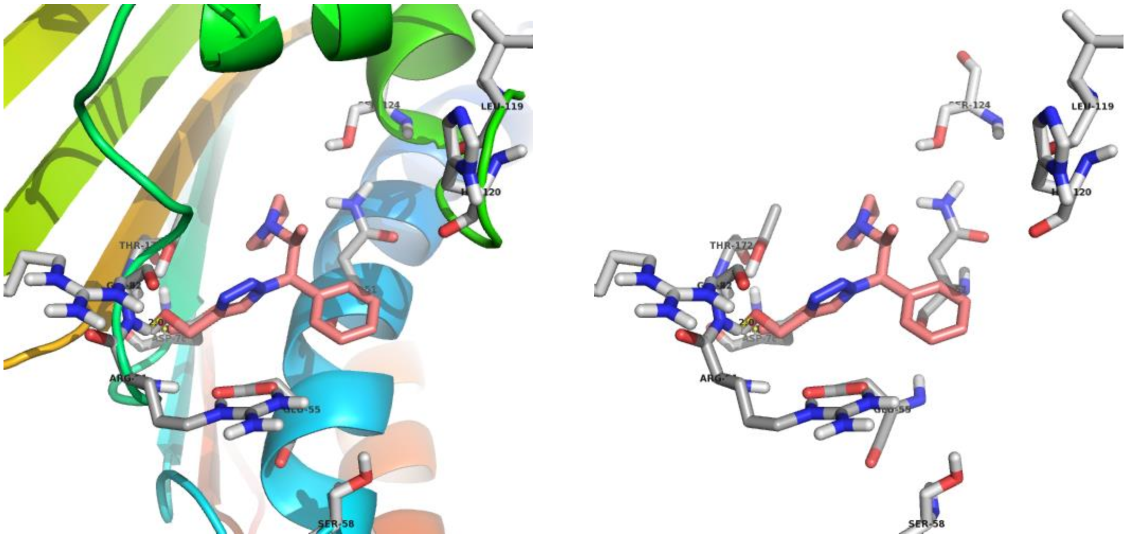

Docking mode of the 5a in the active site of 4EMV

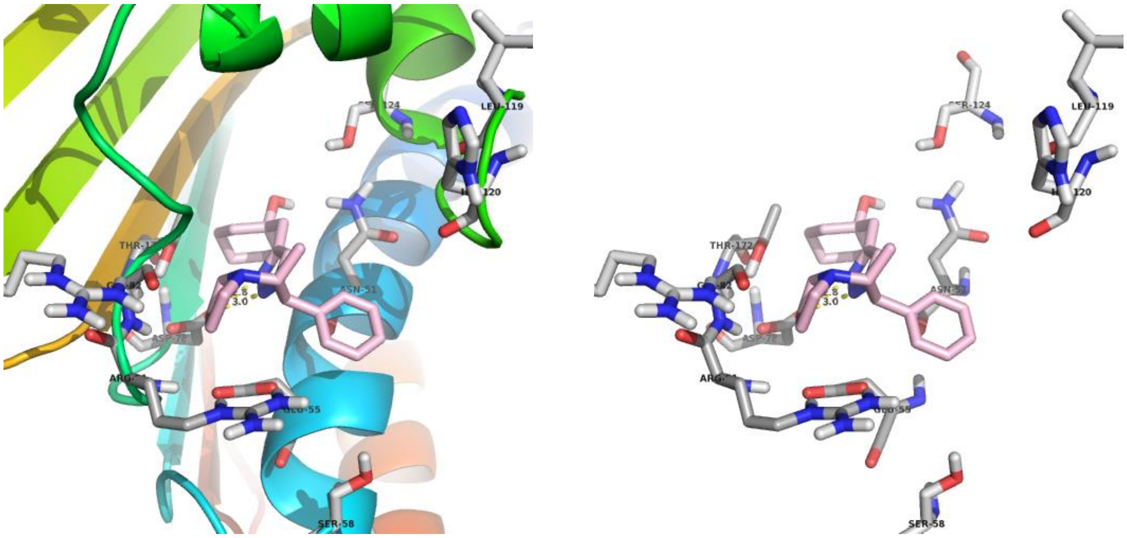

Docking mode of the 5b in the active site of 4EMV

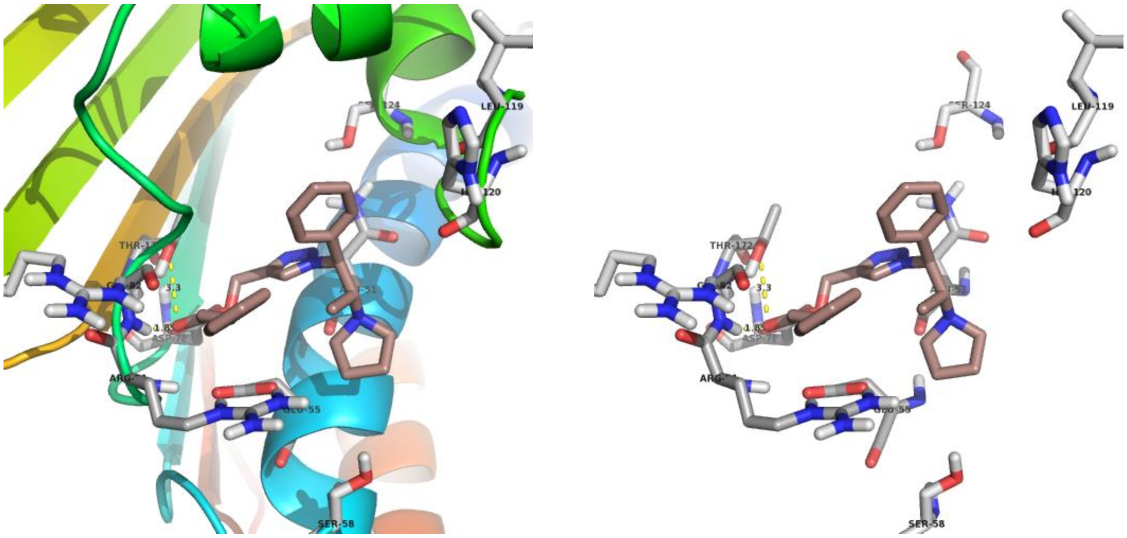

Docking mode of the 5c in the active site of 4EMV

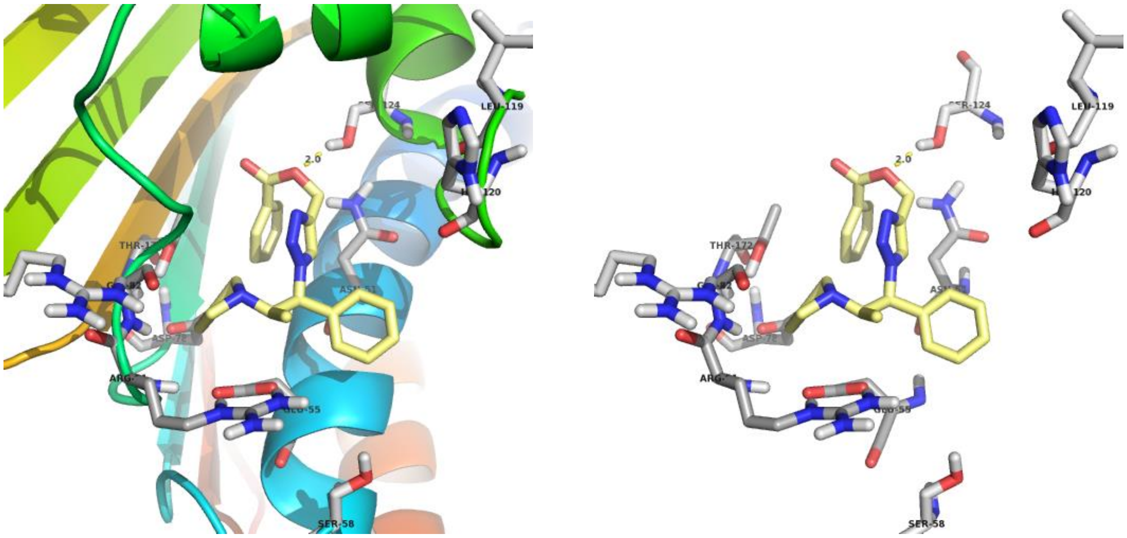

Docking mode of the 5d in the active site of 4EMV

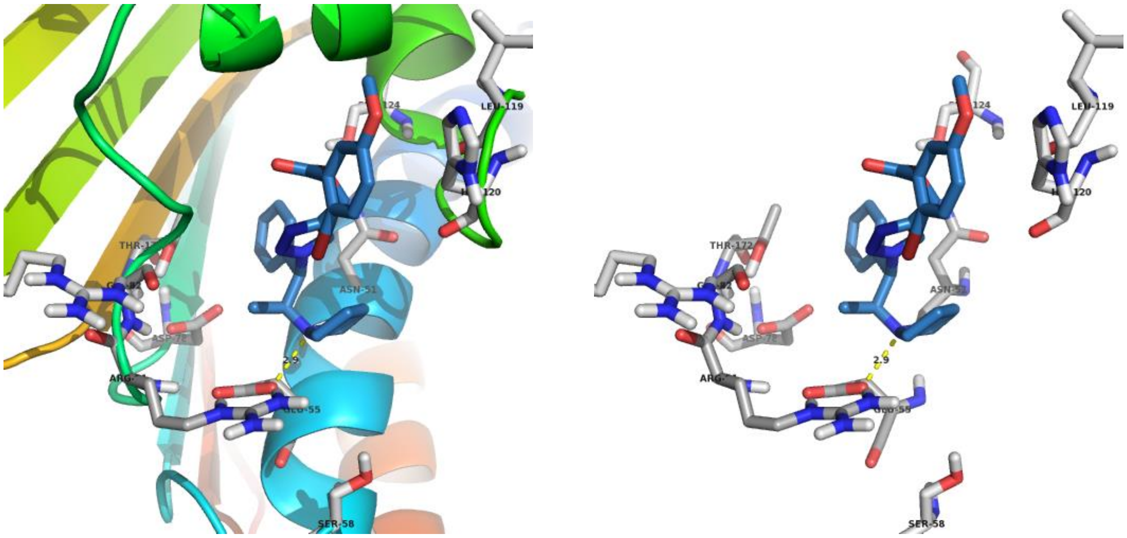

Docking mode of the 5e in the active site of 4EMV

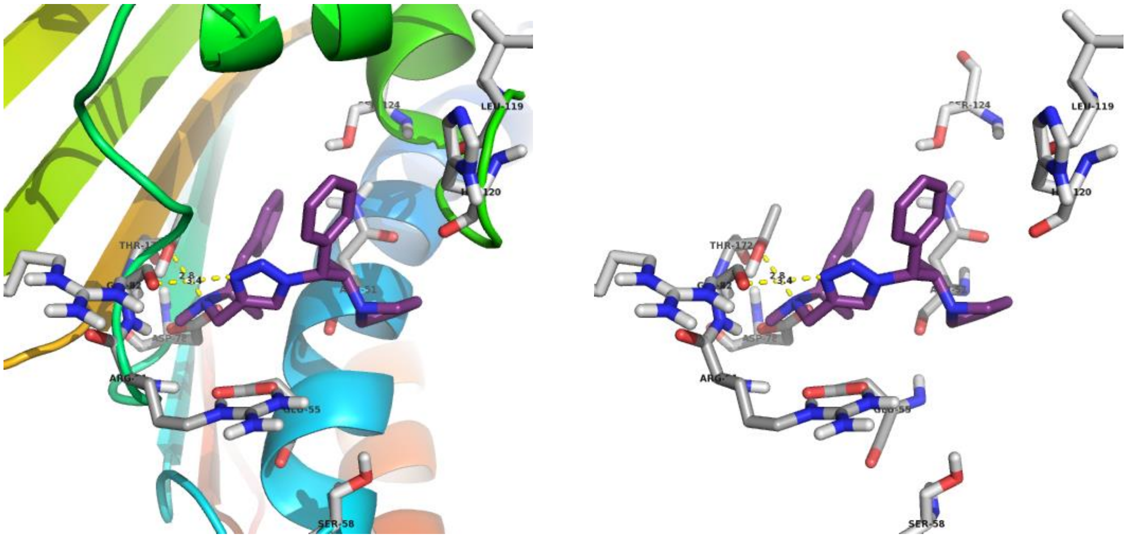

Docking mode of the 5f in the active site of 4EMV

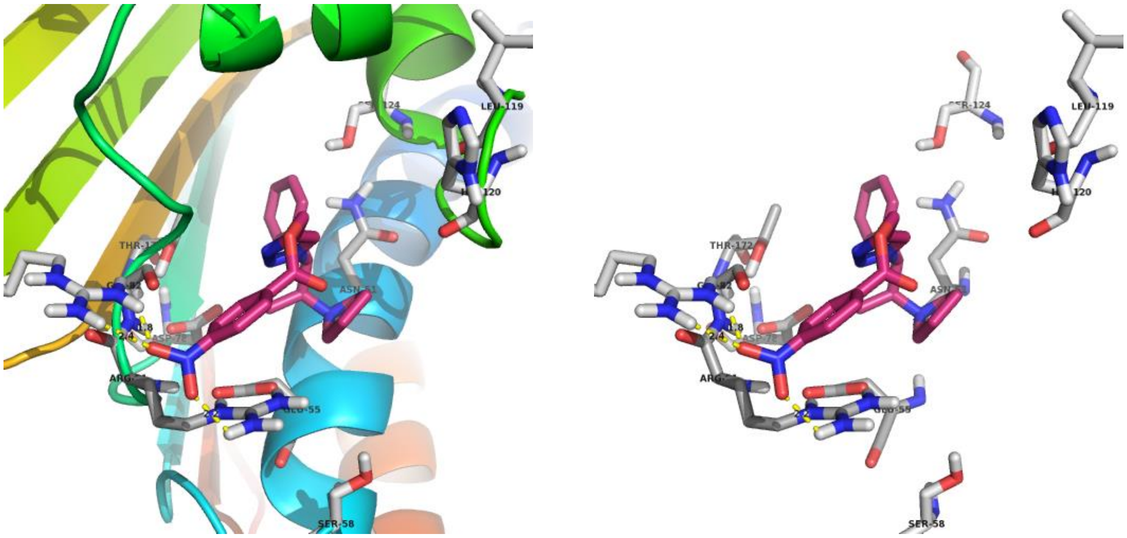

Docking mode of the 5g in the active site of 4EMV

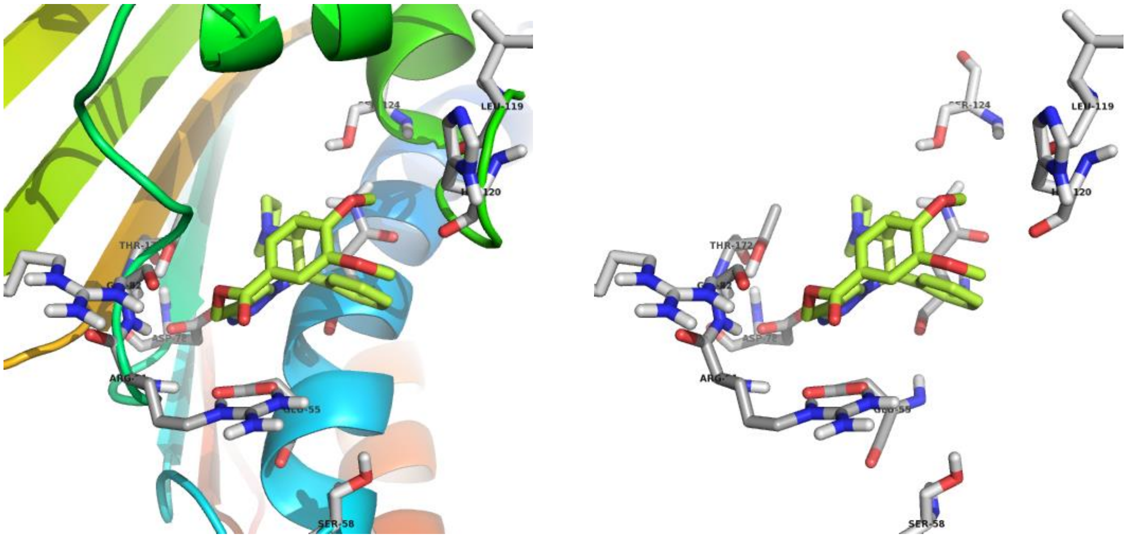

Docking mode of the 5h in the active site of 4EMV

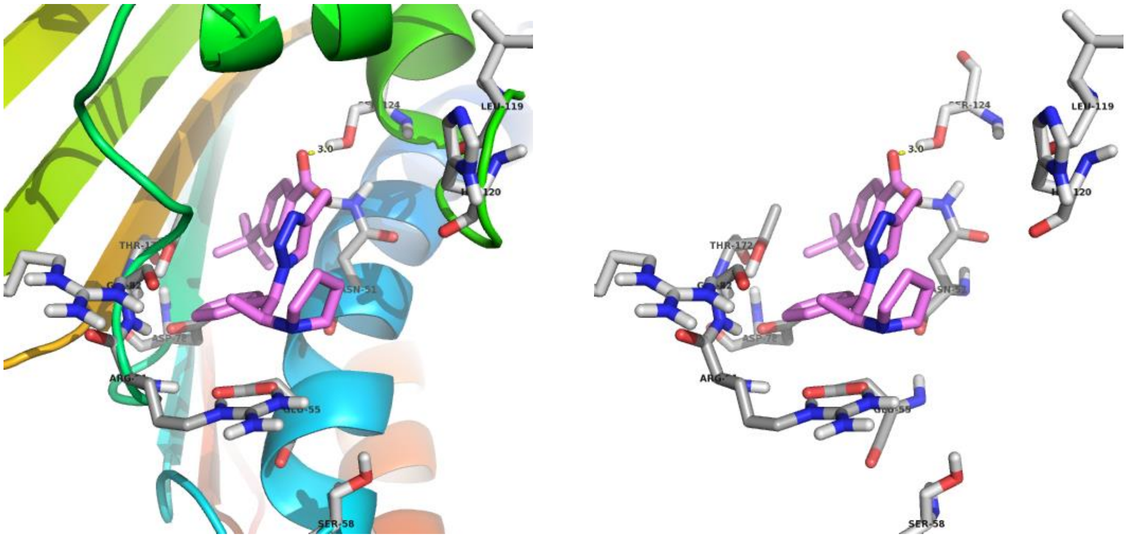

Docking mode of the 5i in the active site of 4EMV

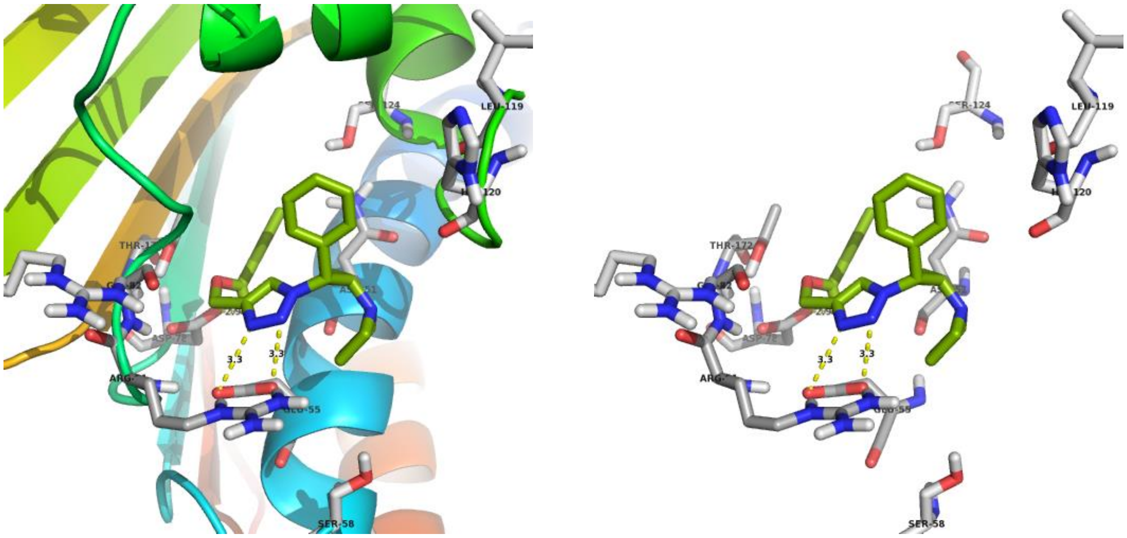

Docking mode of the 5j in the active site of 4EMV

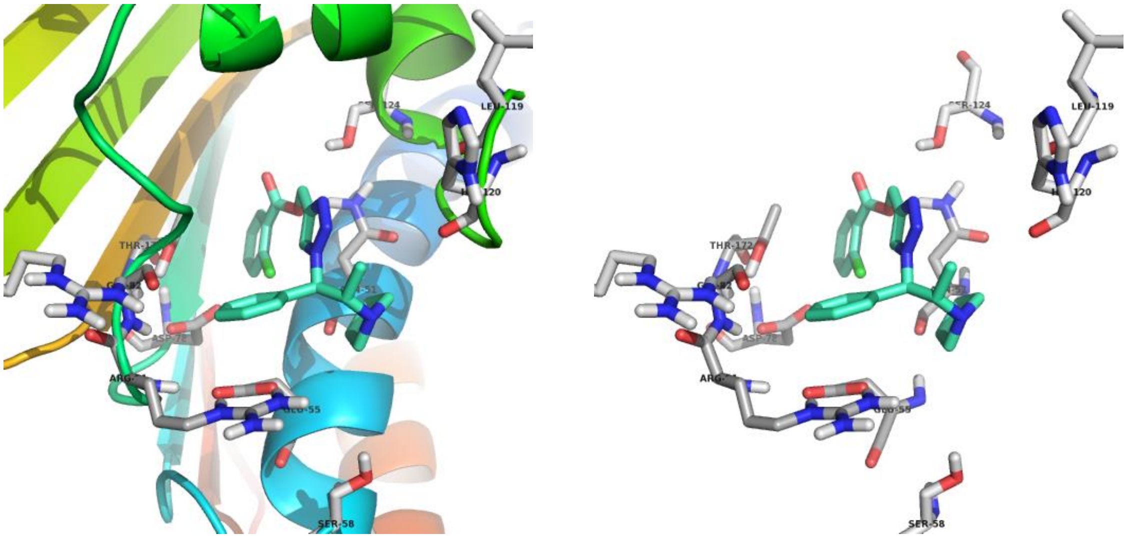

Docking mode of the 5k in the active site of 4EMV

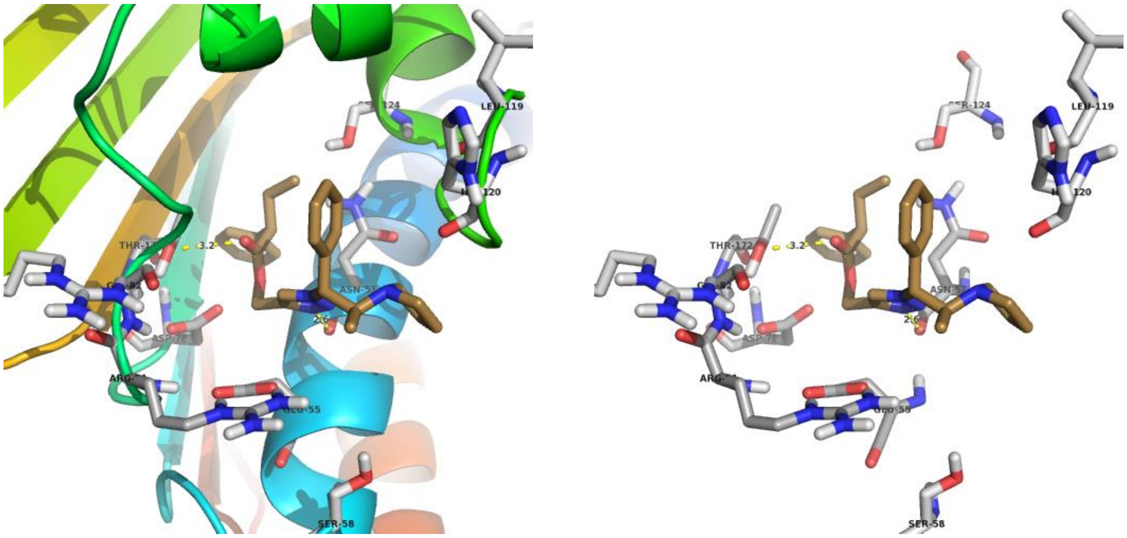

Docking mode of the 5k in the active site of 4EMV

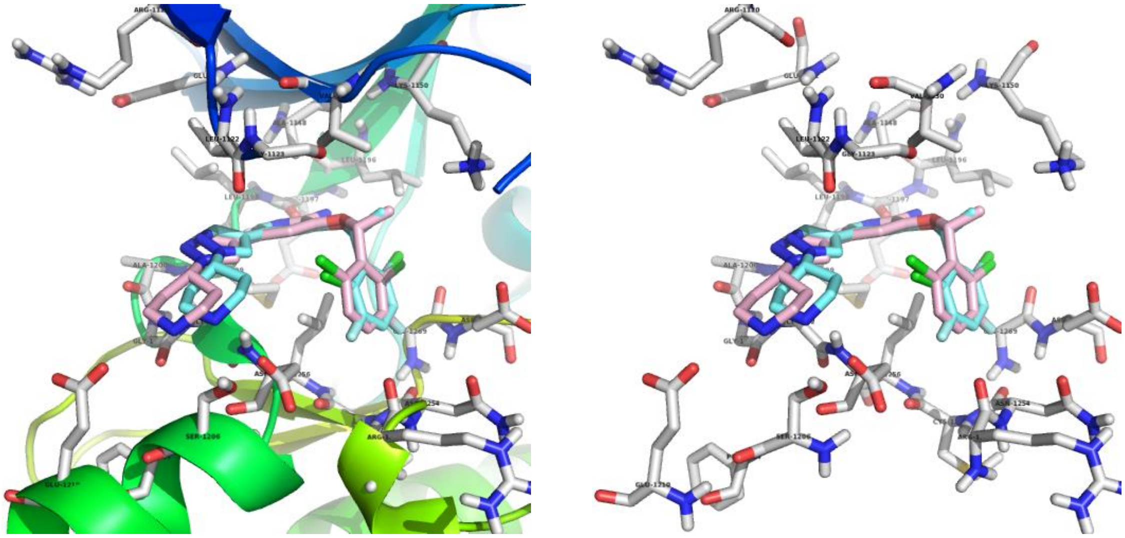

Method validation using crystallised and docked ligand with 2XP2 receptor

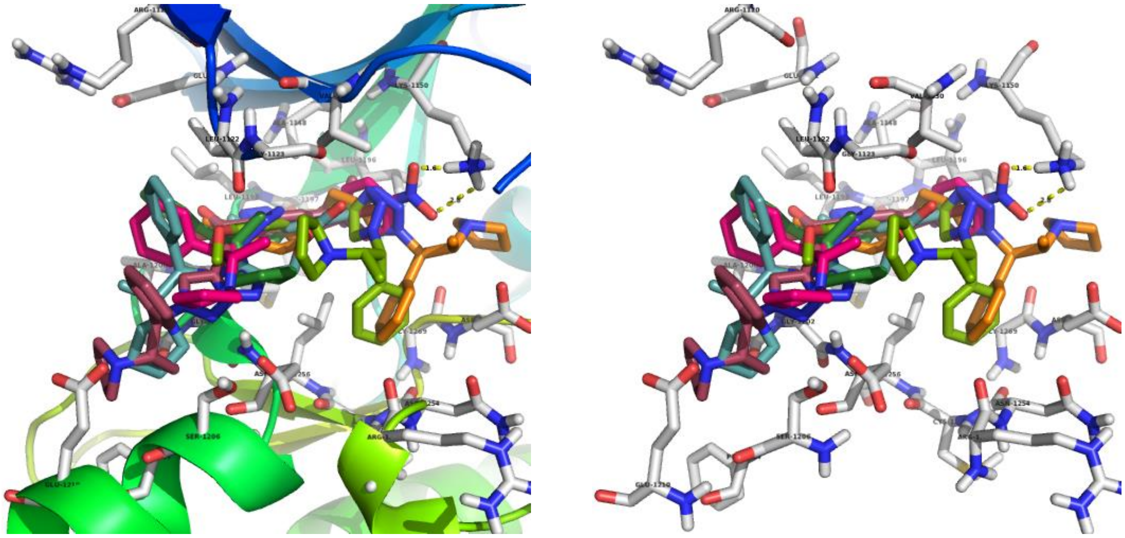

Docking mode of all the compounds in the active site of 2XP2 receptor

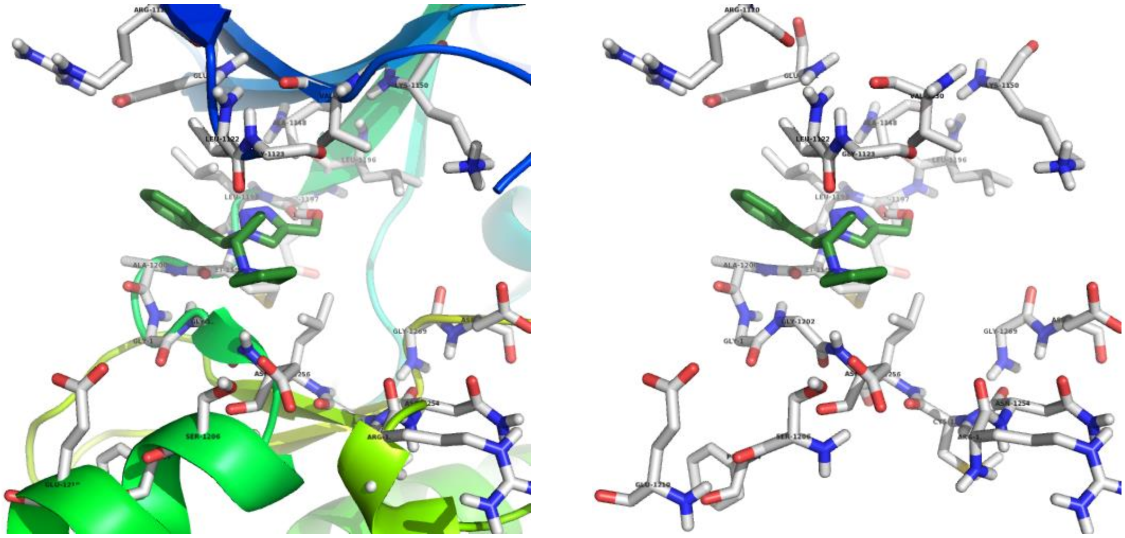

Docking mode of the 5a in the active site of 2XP2

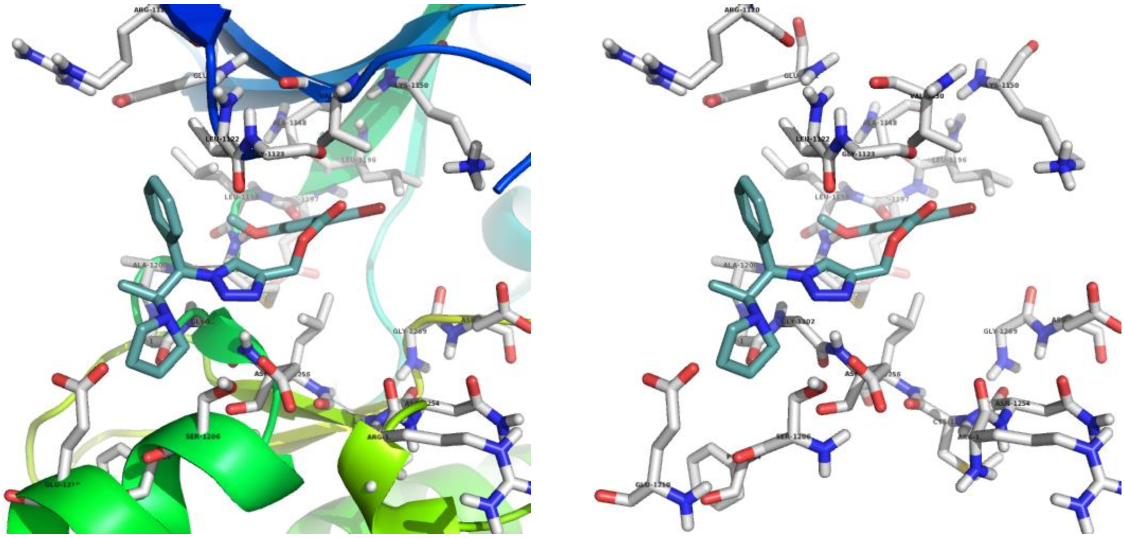

Docking mode of the 5e in the active site of 2XP2

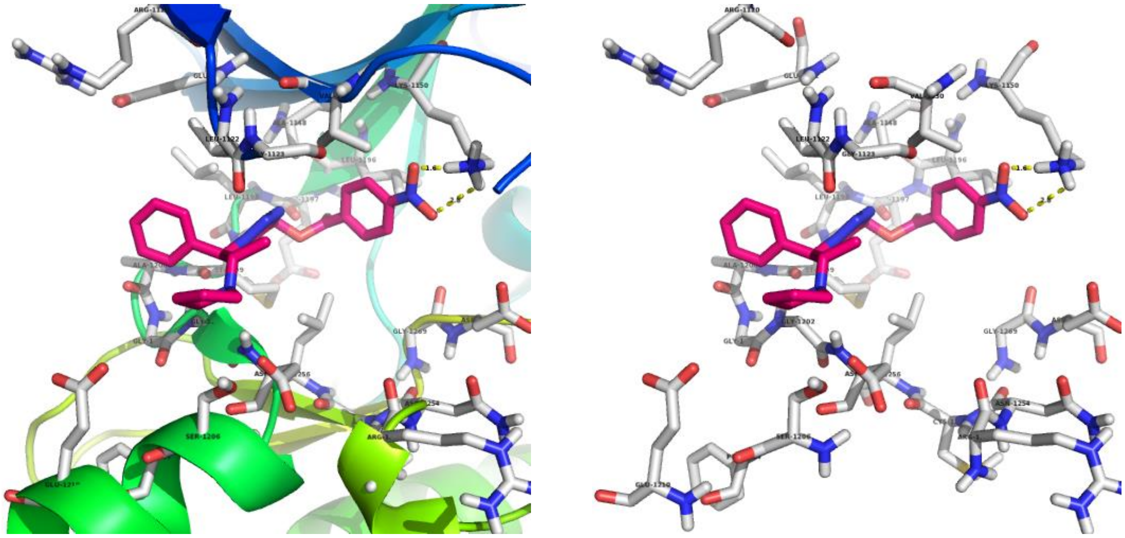

Docking mode of the 5g in the active site of 2XP2

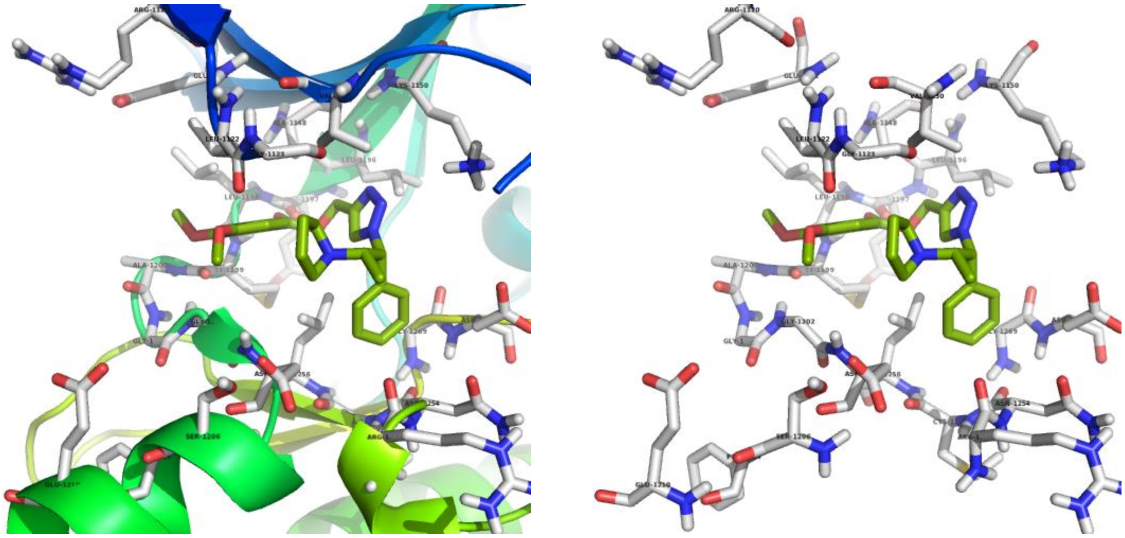

Docking mode of the 5h in the active site of 2XP2

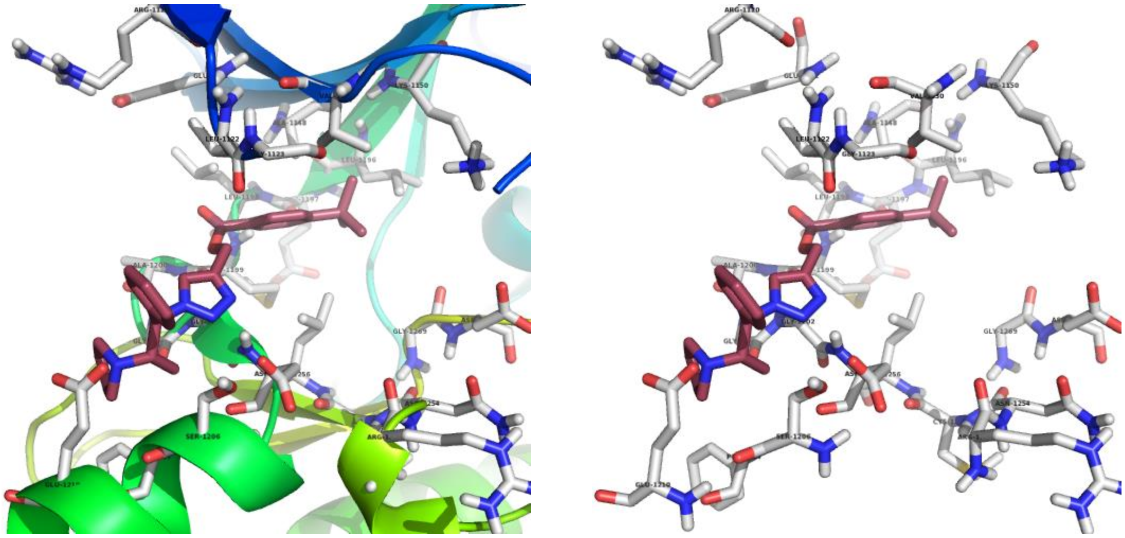

Docking mode of the 5i in the active site of 2XP2

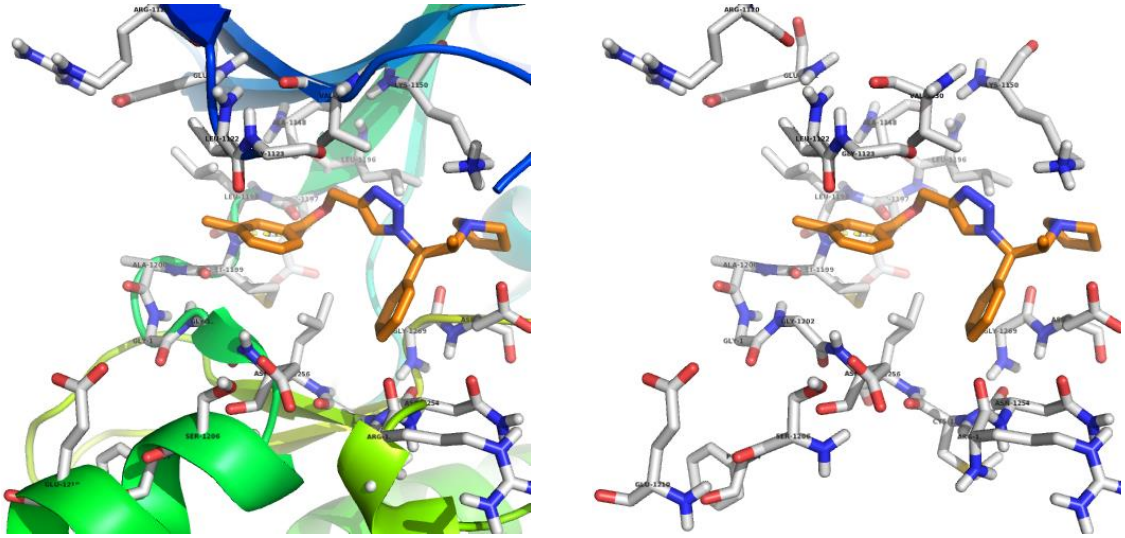

Docking mode of the 5j in the active site of 2XP2

References:

1. (a) Sanner, M.F. *J. Mol. Graphics Mod.* **1999**, *17*, 57. (b) Morris, G.M.; Huey, R.; Lindstrom, W.; Sanner, M.F.; Belew, R.K.; Goodsell, D.S.; Olson, A.J. *J. Comput. Chem.* **2009**, *30*, 2785.

2. Protein Data Bank (http://www.rcsb.org/pdb).
